# Assessing risk factors for malnutrition among women in Bangladesh and forecasting malnutrition using machine learning approaches

**DOI:** 10.1186/s40795-023-00808-8

**Published:** 2024-02-01

**Authors:** Estiyak Ahmed Turjo, Md. Habibur Rahman

**Affiliations:** Department of Statistics and Data Science, Jahangirnagar University, Dhaka, Bangladesh

**Keywords:** Malnutrition, Machine learning, Cross validation, Bangladesh

## Abstract

**Background:**

This paper presents an in-depth examination of malnutrition in women in Bangladesh. Malnutrition in women is a major public health issue related to different diseases and has negative repercussions for children, such as premature birth, decreased infection resistance, and an increased risk of death. Moreover, malnutrition is a severe problem in Bangladesh. Data from the Bangladesh Demographic Health Survey (BDHS) conducted in 2017-18 was used to identify risk factors for malnourished women and to create a machine learning-based strategy to detect their nutritional status.

**Methods:**

A total of 17022 women participants are taken to conduct the research. All the participants are from different regions and different ages. A chi-square test with a five percent significance level is used to identify possible risk variables for malnutrition in women and six machine learning-based classifiers (Naïve Bayes, two types of Decision Tree, Logistic Regression, Random Forest, and Gradient Boosting Machine) were used to predict the malnutrition of women. The models are being evaluated using different parameters like accuracy, sensitivity, specificity, positive predictive value, negative predictive value, $F_1$ score, and area under the curve (AUC).

**Results:**

Descriptive data showed that 45% of the population studied were malnourished women, and the chi-square test illustrated that all fourteen variables are significantly associated with malnutrition in women and among them, age and wealth index had the most influence on their nutritional status, while water source had the least impact. Random Forest had an accuracy of 60% and 60.2% for training and test data sets, respectively. CART and Gradient Boosting Machine also had close accuracy like Random Forest but based on other performance metrics such as kappa and $F_1$ scores Random Forest got the highest rank among others. Also, it had the highest accuracy and $F_1$ scores in k-fold validation along with the highest AUC (0.604).

**Conclusion:**

The Random Forest (RF) approach is a reasonably superior machine learning-based algorithm for forecasting women’s nutritional status in Bangladesh in comparison to other ML algorithms investigated in this work. The suggested approach will aid in forecasting which women are at high susceptibility to malnutrition, hence decreasing the strain on the healthcare system.

## Background

A healthy lifestyle depends on having a good nutritional diet. Malnutrition is a severe circumstance that happens due to the lack of the right amount of all nutrients and energy which the body demands. A major public health issue in the world is malnutrition, which increases the overall illness burden and mortality rates [1]. Bangladesh is an emerging nation. Malnutrition is still a major issue in Bangladesh, particularly for women and children. Undernutrition and being overweight or obese are two major categories of conditions that fall under the umbrella of malnutrition for women. The World Health Organization (WHO) estimates that 190 crore people are globally overweight and underweight adults are 462 billion. Despite the fact that Bangladesh’s malnutrition rate is declining day by day, it remains a serious problem. Women were underweight to a degree of around 19 percent, while women were overweight to a degree of about 24 percent in Bangladesh [2]. As underweight and overweight are connected with various contagious illnesses such as heart disease, diabetes, stroke, respiratory problems, and cardiovascular disease [3–7]. Underweight women face a number of challenges, including decreased productivity at work and increased risk of spontaneous abortion, low birth weight, intrauterine fetal death, and infant mortality [8, 9]. Melchor et al. (2019) suggested that at the time of pregnancy, delivery, and the postpartum period, maternal obesity can lead to several stumbling blocks for both the mother and the fetus [10]. Various factors contribute to malnutrition. Malnutrition is more likely to occur if risk variables are identified too late or not prominently enough. It’s crucial to identify malnutritional factors early so that women are protected from the diseases that are exacerbated by those. Work must be done to stop malnutrition and identify risk factors sooner. In order to forecast Bangladesh’s malnutrition status and identify the causes of malnutrition, this project is eager to get started.

The health of a person can be determined by his nutritional state. Previous studies revealed many factors that are accountable for malnutrition. Numerous statistical methods such as univariate regression, multivariate regression, logistics regression, Pearson’s correlation, and so on have been proposed to ascertain the causes and consequences of malnutrition [11–24]. Machine learning algorithms are particularly popular to envisage the risk of any diseases accurately [25]. A variety of statistical methods are used by machine learning algorithms to find patterns in big, complex data sets that are relevant depending on prior knowledge [26]. Machine learning is an effective strategy for identifying unknown links or patterns by fusing statistical learning and artificial intelligence [27]. While compared to traditional statistical models, machine learning algorithms have been demonstrated to be more accurate in predicting outcomes when used to solve classification issues. There have already been several research on the topic of predicting nutritional status and finding their factors using the machine learning (ML) approach. Multiple illnesses have been predicted using ML systems such as anemia [28, 29], acute appendicitis [30], cardiovascular defect [31], covid 19 [32, 33], diabetes [27, 34–37], hypertension [38], low birth weight [39–42] utilizing a variety of demographic and health survey datasets as well as common risk factors of the diseases. Several studies had been conducted previously based on malnutrition using machine learning approaches. Talukder and Ahammed (2020) used BDHS, 2014 data for predicting the malnutrition of under-five children in Bangladesh using machine learning techniques. The researchers conclude that Random Forest (RF) is the best algorithm among others [43]. A study that used the Ethiopian Demographic and Health Survey 2016 data carried out by Bitew et al. (2020) found that the Extreme Gradient (xgbTree) algorithm gave a better result [44]. Use of artificial intelligence on the Indian Demographic Health Survey dataset 2005-2006 to identify the likelihood correlation with malnutrition done by Khare et al. (2017) [45]. A study used the Fisher Exact Test to identify the key nutritional determinants in a cross-sectional study [46]. Shahriar et al. (2019) studied Bangladeshi children using ML approaches for classification and discovered that Artificial Neural Network (ANN) was the most effective [47]. Markos et al. (2014) used data mining techniques such as Decision tree (DT), PART rule induction classifiers, and Naïve Bayes found that PART rule induction gave the best accuracy of the result [48]. Howsoever, there isn’t much research that takes ML algorithms into account for predicting malnutrition in women. Research on BDHS 2014 data for predicting malnutrition among women was conducted by Islam et al. (2022) and the result gave some prominent factors and Random Forest (RF) algorithm as the best algorithm to identify malnutrition status among women [3]. Identification of the most influential factors of malnutrition with the help of decision rules, K-nearest neighbor (kNN), Support Machine Vector (SVM), Decision tree (DT), and Bayesian networks was made by Reis et al. (2017) [49]. Momand et al. (2020) suggested that Random Forest (RF) and PART provided the most accurate prediction of malnutrition based on their study [50]. Rahman et al. (2021) reveal that LR-RF based combinedly is able to classify and forecast stunted, wasted, and underweight children with greater accuracy [51].

Consequently in order to envisage the risk of women’s malnutrition in Bangladesh, many ML algorithms that had not been widely employed in earlier studies are going to apply in the following study. The focus of the current work is to identify possible risk factors for malnutrition in women and to fit models for different ML algorithms. This research provides strong predictive models that improve our understanding of this important health issue and give healthcare professionals and policymakers useful information for focused responses. Incorporating machine learning forecasts into existing health systems can enhance their overall capacity for monitoring, analyzing, and addressing malnutrition. The ultimate goal of this study is to use its findings to spur wise decisions that will help Bangladeshi women live longer and in better health. It will also be used to determine the most accurate model for forecasting the risk of malnourishment.

## Methods

Ergonomic assessments will be used in the BDHS, 2017-18 to examine women’s nutritional status. The last chapter discussed the previous study and the background and motivation of this study. Within this following chapter, the methods that are used to evaluate women’s malnutrition are described. The possible risk factors were obtained by Chi-square and then used six machine learning approaches Naïve Bayes (NB), Classification and Regression Tree (CART), C5.0 Classification (C5.0), Logistic Regression (LR), Random Forest (RF), and Gradient Boosting Machine (GBM) to predict malnourishment among the women. To determine the best predictive model, the performance of these ML approaches was evaluated using evaluation parameters. The national research committee’s ethical principles were followed in all procedures concerning human participation in this study.

*Dataset:* The Bangladesh Demographic and Health Survey (BDHS) 2017-18 is the source of the data used in this study. The Bangladesh Demographic and Health Survey (BDHS) 2017-18 is the latest and eighth nationwide survey to provide information on the demographic and health status of women and children [52]. The sampling frame for the 2017-18 BDHS is premised on the Bangladesh Bureau of Statistics’ detailed list of enumeration areas (EAs) encompassing the entire country, which was prepared for the People’s Republic of Bangladesh’s population census in 2011. A two-stage stratified sample approach is used in the survey to choose respondents. Each division was further subdivided into urban city corporations, urban areas other than city corporations, and rural areas, for a total of 22 sampling strata. During the initial stage, 675 EAs were chosen with a probability proportional to EA size and independently in each sampling stratum, in which 277 were from cities and 448 were from rural areas. In the second phase of the selection process, a total of thirty households per cluster was chosen at the systematic selection from a newly established household listing with an equal probability. Based on this sampling technique they selected 20,250 households and about 20,108 ever-married women between the ages of 15 to 49 who are regular members of the sample households. These individuals are the representative of the women population of Bangladesh and face-to-face interview sessions were carried out with them [52]. A total of 17,022 individuals are selected for ultimate analysis after excluding missing values, unusual observations, and do not know. The body mass index(BMI) acknowledge as the response category and WHO categorized it as underweight (BMI < 18.5), normal (18.5 $\le$ BMI $\le$ 24.9), overweight (25.0 $\le$ BMI < 30.0), and obese (BMI > 30.0) [53]. The unit of these categories is $kg/m^2$. Underweight, overweight, and obese are merged as a single category of malnutrition for women. The factors are chosen for this study according to past studies [3, 21, 54].

*Naïve Bayes:* Bayesian classification is founded on Bayes’ theorem, named after Thomas Bayes, who pioneered work in probability and decision theory in the 18th century. Bayesian classifiers are capable of anticipating probabilities of class membership, such as the likelihood that a specified tuple falls within a particular class [55]. Naïve Bayes is a straightforward probabilistic model that really can manage relatively high data with ease [56]. The Naïve Bayes estimates the tuple like as X belongs to the class C by optimizing the posterior probability P(C|X) with the support of prior probability P(C) and P(X) and conditional probability P(X|C) which can be demonstrated as,

1 $$\begin{aligned} P(C|X)=\frac{P(X|C) \times P(C)}{P(X)} \end{aligned}$$

P(X|C) and P(C) are usually required to be emphasized since P(X) is consistent throughout all classes. ${P(X_i|C_i)\times P(C_i)}$ is calculated for every category ${C_i}$ to forecast the class label of X. The classifier predicts that perhaps the class label of tuple X is the class Ci if and only if,

2 $$\begin{aligned} {P(X_i|C_i)P(C_i)}>{P(X_j|C_j)P(C_j)} \end{aligned}$$

The anticipated class label corresponds to the class ${C_i}$ for which ${P(X_i|C_i)}$ is maximum.

*Decision Tree Induction:* Decision tree (DT) learning is a form of supervised learning that has applications in data mining, statistics, and machine learning. Throughout the late seventies and early eighties, J. Ross Quinlan, a researcher in machine learning, created the decision tree method which is known as Iterative Dichotomiser also said ID3 [55]. Later C4.5, the successor of ID3 and the Classification and Regression Tree is developed as a form of the decision tree. Han et al. (2012) also quoted that ID3, C4.5, and CART employ an optimization technique related to a nonbacktracking approach wherein decision tree algorithms are built inside a top-down recursive divide-and-conquer fashion [55]. The methodology followed by DT which is suggested by Han et al. (2012) have some steps [55]. (i) The algorithm is invoked by passing three parameters: The dataset, the attribute list, and the Attribute selection method; (ii) The tree begins with a single leaf, N, which represents the tuples of training in D; (iii) If all of the tuples in D belong to the same class, leaf N will be categorized with the class; (iv) Else the algorithm uses the Attribute selection approach to establish the splitting criterion; (v) The splitting criterion is designated at node N, and it acts as a test at the node. For each conclusion of the splitting criterion, a branch is formed from node N; (vi) The method recursively applies the same technique to create a decision tree for the data items for every resulting partition; (vii) The recursive partitioning process is terminated immediately if all of the itemsets in subdivision D are members of the same class, or there aren’t any additional features that allow the tuples can be further divided up, or there are also no tuples for a certain branch, hence partition Dj is empty; (viii) The eventual results decision tree is restored.

*(a) Classification and Regression Tree:* L. Breiman, J. Friedman, R. Olshen, and C. Stone, a group of statisticians in 1984, publicly released the book Classification and Regression Trees (CART) [57]. CART is one kind of decision tree induction. The procedure of CART is the same as DT and it uses the Gini index as an attribute selection method for splitting effectively a given set of data partitions. The Gini index quantifies the contamination of D, which might be a data partition or a set of training tuples. It measures by

3 $$\begin{aligned} Gini(D) = 1 - \sum \limits _{i=1}^{n} p_i^2 \end{aligned}$$

Here, p is the probability of tuple D. Gini(D) gives the partitioning as,

4 $$\begin{aligned} Gini_A(D)= \frac{|D_1|}{|D|} \times {Gini (D_1)} + \frac{|D_2|}{|D|} \times {Gini (D_2)} \end{aligned}$$

And finally,

5 $$\begin{aligned} \Delta Gini(A) = Gini(D) - Gini_A(D) \end{aligned}$$

The splitting property is chosen to maximize impurity reduction. The procedure continues till the data can no longer be split any further. CART is capable of handling both data types, numerical as well as categorical.

*(b) C5.0:* J. Ross Quinlan, a computer scientist, established the C5.0 algorithm as an improvement on his preceding algorithm, C4.5 [58]. C5.0 classification is the best-known decision tree that divides data according to the subject matter which generates the most information gain. The splitting factor is chosen by the highest information gain of the attribute. Each subset represented through the initial partition is again partitioned, typically relying on a different subject, as well as the procedure is carried out until the subsets can neither be separated further. When particularly in comparison to others, C5.0’s decision trees operate similarly yet are substantially simpler to comprehend and implement. It can handle both quantitative and qualitative criteria.

*Logistic Regression:* Logistic regression is a method of supervised learning that transforms the outcome of a linear model to ensure it conforms to a binary response. It is frequently regarded as the most widely utilized machine learning procedure for dealing with binary classification. To assess the attribute of concern, LR engages in the estimation of the maximum likelihood process. Logistic regression is commonly used in the case of a combination of one binary response variable and one or more quantitative predictor variables that have been related to the probability or odds of the dependent variables [59]. For instance, if $Z_i$ are the n number of factors, then the logistic model can be expressed as

6 $$\begin{aligned} p(z) = \frac{exp(\alpha _0+\beta Z_i)}{1+exp(\alpha _0+\beta Z_i)};i=1,2,...,n \end{aligned}$$

It also can be expressed by

7 $$\begin{aligned} log \left(\frac{p(z)}{1-p(z)}\right)= exp\left(\alpha _0+\beta Z_i\right) \end{aligned}$$

The probability of an event occurring is represented by *p*(*z*), while the probability of an event not occurring is represented by $1-p(z)$.

*Random Forest:* Random Forest is one of the most influential algorithms, constructed in 2001 by L. Breiman [60]. It is a categorization method that relies on the development of a group of tree-structured classifiers. The RF algorithm chooses the number of feature factors at irregular intervals and builds a decision tree from them. The procedure of CART is applied for building the trees [55]. RF works as Hastie et al. (2009) stated [61] as (i) First, the algorithm creates bootstrapping samples Z of the total size of the training data N from a training set with a replacement which is also known as bagging; (ii) Expand the random-forest tree towards the bootstrap sample by recursively repeating the procedures below for each tree terminal node until the minimum node size is attained - (a) Among the p variables, choose m variables at random, (b) Choose the best variable or split-point from the m options, (c) Divide each node into two daughter nodes; (iii) Return the tree ensemble. This is how the RF works to build up a model. Then the model is used for further classification of the new data set or test data set.

*Gradient Boosting Machine:* Gradient Boosting Machines (GBM) are one of the most well-known boosting methods developed by Jerome Friedman [62]. GBM is thought to be on par with high-performance methods such as random forests. GBM works similarly to RF, though the trees are formed successively and then each tree utilizes knowledge from previously developed trees [59]. GBM starts with the initial leaf and further constructs the trees based on the pseudo residuals of the previous trees. Also, every tree is fitted to a distinct rendition of the initial set of data. GBM is one kind of boosting method as it creates sequential trees and the final tree has the highest accuracy. All the constructed trees are combined to yield a single predictive model that is utilized for further envisage.

*Algorithm Assessment:* These 6 algorithms are evaluated with evaluation parameters such as accuracy (Ac), sensitivity (SE), specificity (SP), positive predictive value (PPV), negative predictive value (NPV), cohen’s Kappa, $F_1$ score, and roc curve and AUC in this study. Furthermore, k-fold cross-validation techniques are used and determine these performance measures. The confusion matrix is constructed for all the classifiers that consist of true positive (TP), true negative (TN), false positive (FP), and false negative (FN). This matrix makes it possible to quantify these evaluation parameters [63].

*Accuracy* of any predictive method is the foundation for assessing its performance. It calculates the proportion of correctly predicted overall data points evaluation. The best possible accuracy is 1.0, while the lowest possible accuracy is 0.0. It is simple to compute by dividing the number of correctly predicted by the grand total of projections. Also, it can be expressed as,

8 $$\begin{aligned} Accuracy = \frac{TP+TN}{TP+FP+TN+FN} \end{aligned}$$

This study compiled the best levels of accuracy achieved through different ML algorithms.

*Sensitivity* refers to the model’s ability to correctly identify those who are genuine positive cases. It is also referred to as recall or true positive rate. Sensitivity is determined by dividing the complete number of positive aspects by the number of true-positive outcomes also includes false positives. Sensitivity can be calculated mathematically as follows

9 $$\begin{aligned} Sensitivity = \frac{TP}{TP+FN} \end{aligned}$$

*Specificity* refers to the model’s capacity to properly recognize those who are the true negative cases. It is also referred to as a true negative rate. The number of true-negative results is divided by the entire amount of negatives to quantify specificity which also includes false negatives. Specificity can be expressed as

10 $$\begin{aligned} Specificity = \frac{TN}{FP + TN} \end{aligned}$$

*Positive predictive value* refers to a model’s ability to predict the presence of positive cases among those who predicted positive. Precision is another term for it. This trait can anticipate how likely someone is to be a true positive case in the event of a positive test result. PPV can be measured by

11 $$\begin{aligned} Positive \ Predictive \ Value = \frac{TP}{TP + FP} \end{aligned}$$

*Negative predictive value* is the ability of a model to foresee the existence of negative cases in those who expected negative. The negative predictive value is a metric used to assess how accurate a particular model is. Mathematically, NPV can be calculated as follows

12 $$\begin{aligned} Negative \ Predictive \ Value = \frac{TN}{FN+TN} \end{aligned}$$

*Cohen’s Kappa* statistic is a more effective approach for dealing with multi-class and misaligned class problems. It is the ratio of comprehension among both anticipated and actual classification in a set of data. The Kappa statistic can be utilized to assess not only a single classifier but multiple classifiers in conjunction. The statistic’s values provide the following information: 0 implies no agreement, 0 to 0.20 as slight, 0.21 to 0.40 as fair, 0.41 to 0.60 as moderate, 0.61 to 0.80 as substantial, and 0.81 to 1 as almost perfect [64]. It can be measured by

13 $$\begin{aligned} Kappa = \frac{{Accuracy - Random \ Accuracy}}{1-Random \ Accuracy} \end{aligned}$$

where,

14 $$\begin{aligned} Random \ Accuracy=\frac{(TN+FP)\times (TN+FN)+(FN+TP)\times (FP+TP)}{(TP+FP+TN+FN)\times (TP+FP+TN+FN)} \end{aligned}$$

The $F_1$ score is a way of measuring a test’s accuracy. It is determined by the test’s precision and recall. Precision is also referred to as positive predictive value, while recall is referred to as sensitivity. The $F_1$ score determines how often a model is predicted correctly throughout the entire data. The harmonic mean of a model’s precision and recall is used to calculate an $F_1$ score. The following equation below can be used to measure it

15 $$\begin{aligned} F_1 = \frac{2 \times Precision \times Recall}{Precision + Recall} \end{aligned}$$

The *ROC curve* is also known as the receiver operating characteristic curve. It is a graph that depicts a classification model’s performance across all classification thresholds. This curve depicts two parameters, true positive rate, and false positive rate. AUC is an abbreviation for the area under the ROC Curve. AUC calculates the area beneath the entire ROC curve in two dimensions. AUC compares two models and assesses the efficiency of the same model throughout different thresholds.

## Results

The Bangladesh Demographic and Health Survey (BDHS) 2017-18 is the eighth nationwide study to offer information on women’s demographics and health conditions and this information is used in this study to predict the malnutrition of women. A total of 1227 variables are in the information of women and among them, 15 variables including the response variable are taken. The models described in the last chapter are being used in this data to apprise the nutritional situation of women. The data that has been collected from BDHS, 2017-18 is discussed in this section of the research work. It gives a proper idea about the whole set of data and comprehends the summary of data.

The study is based on envisaging malnourishment among women in Bangladesh. The data for this study came from the Bangladesh Demographic and Health Survey BDHS, 2017-18. The body mass index (BMI) is a measurement that employs weight and height to work out if the weight is fit and active, so BMI is chosen as the explained variable. 14 exposure variables are taken considering the past studies [3, 21, 54]. Table 1 below contains a list .

**Table 1:** Factors classification of malnutrition used in this study

Factors	Description	Factor types	Class level
Age	Age in group	Categorical	15-24, 25-34, 35-49
Division	Division	Categorical	Barisal, Chittagong, Dhaka, Khulna, Mymensingh, Rajshahi, Rangpur, Sylhet
Residence	Type of place of residence	Categorical	Urban, Rural
Wealth index	Wealth index	Categorical	Poor, Middle, Rich
Education	Highest educational level	Categorical	No education, Primary, Secondary, Higher
Currently working	Respondent currently working	Categorical	No, Yes
Children ever born	Number of children ever born	Categorical	1-2, 3-4, 5 or more
Birth in last 5 years	Number of birth in last 5 years	Categorical	1-2, 3-4
Currently pregnant	Pregnancy status	Categorical	No, Yes
Currently breastfeeding	Breastfeeding status	Categorical	No, Yes
Husband’s Education	Highest educational level	Categorical	No education, Primary, Secondary, Higher
Drinking water	Source of drinking water	Categorical	Safe, Unsafe
Toilet facility	Type of toilet facility	Categorical	Hygenic, Unhygenic
Cooking fuel	Type of cooking fuel	Categorical	Improved, Unimproved

**Fig. 1:**
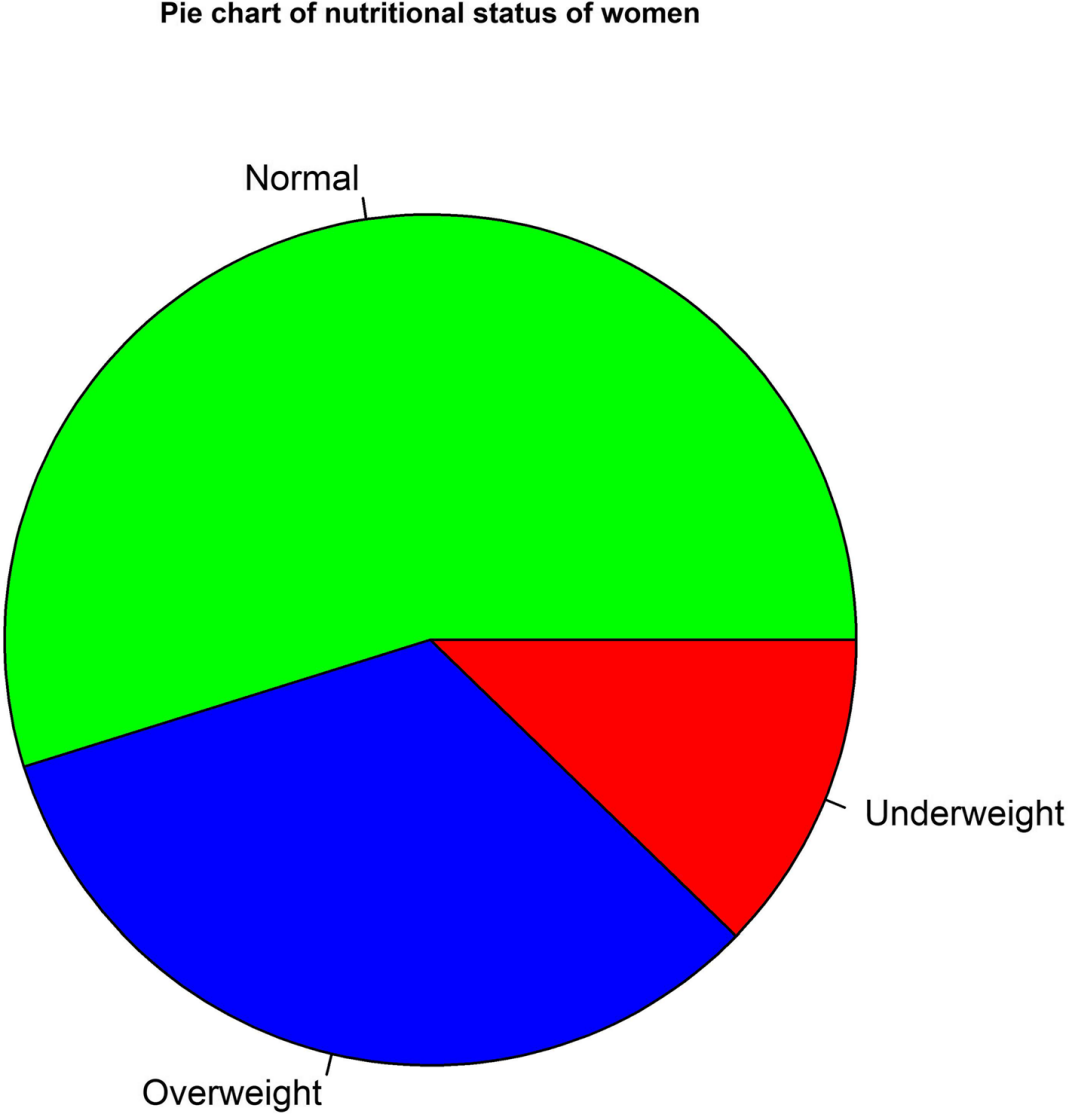
Pie chart of the nutritional status of women in BDHS, 2017-18

The study is conducted with the individuals of 17022 in total from BDHS, 2017-18 after excluding all irrelevant data. The value of BMI for these women is classified into four types such as underweight, normal, overweight, and obese. Overweight and obese can be treated in the same category. The BMI of the women tells their nutritional status of them. Figure 1 represents the present nutritional situation of women who are selected for the study. Around 50 percent of the individuals are healthy. Nearly half of the women are facing malnutrition problems and among them, the majority are suffering from being overweight.

Underweight and overweight have been combined as the category of malnutrition for this study. So the experimental variable has two categories for this study, normal and malnutrition. The data set includes 7691 malnourished women, representing 45.18 percent of the total population. On the other side, 54.82 percent of individuals in the study data set are normal that includes 9331 women.

**Fig. 2:**
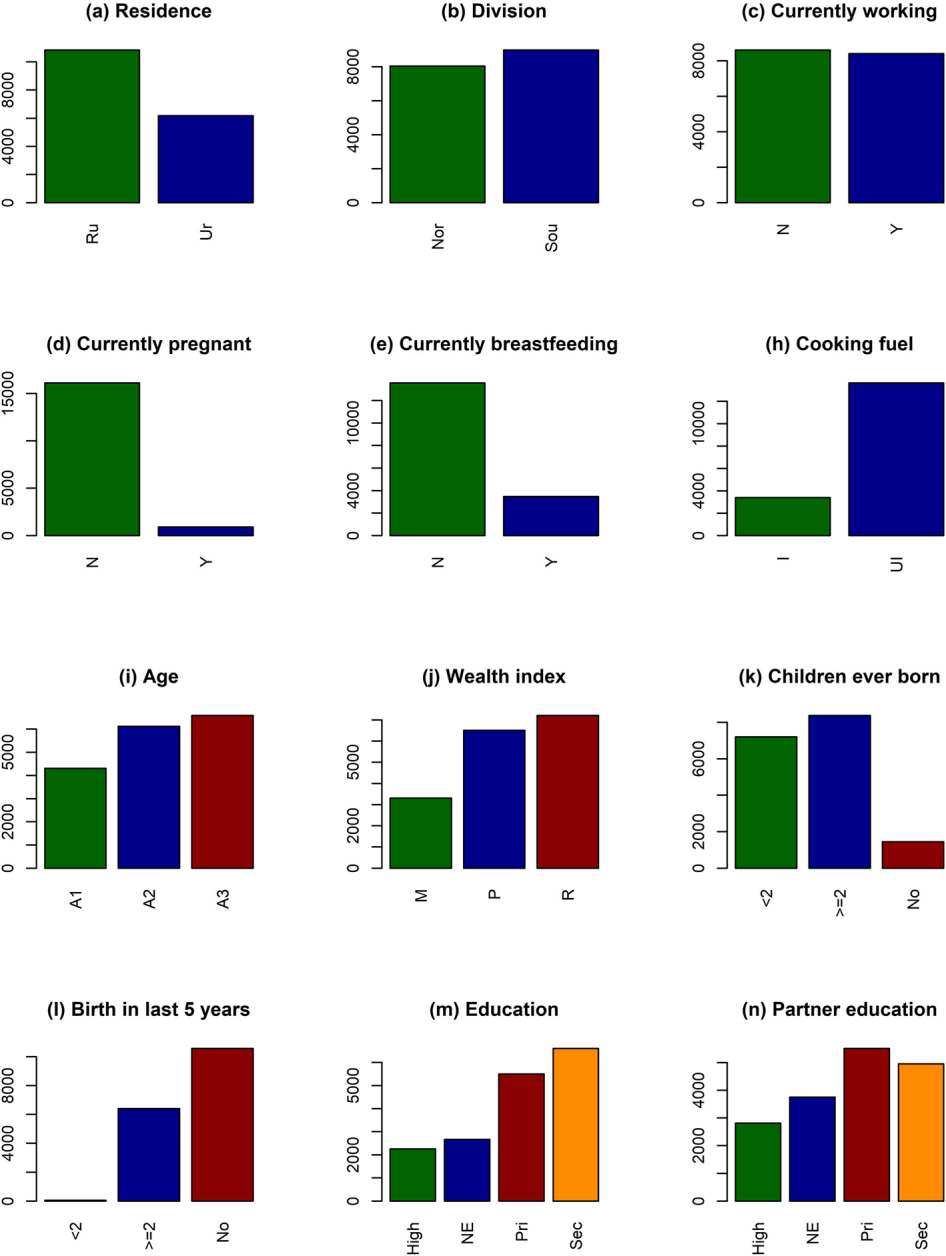
Bar chart of different factors related to respondents for this study

The variables which are considered as the risk factor are summarized in Fig. 2 and Table 2. There are 14 risk factors in the study. There are two types of residence, urban and rural from where the respondents came from. The majority of the women are from rural areas. About 63.68 percent of individuals which consist of 10839 women are from rural areas, and the rest of the women, nearly 36.32 percent are from urban areas. Bangladesh is divided into eight divisions. For the purposes of the study, the divisions are merged into two categories: south and north. The south part contains Barisal, Chattagram, Dhaka, and Khulna and on the other side, the north part has Mymensingh, Rajshahi, Rangpur, and Sylhet. The individuals from the south and north are 8981 and 8041 respectively. All the respondents are ever-married women from the age of 15 to 49. This age interval has been divided into three categories. A1 is from 15 to 24, A2 is from 25 to 34, and A3 is from 35 to 49. Most of the women are from A2 and A3 groups, about 35.99 and 38.69 percent, and lesser from the A1 group. The wealth index is a variable with three sections, and many of those surveyed come from rich families. A total of 7205 women are from the rich section whereas 6510 and 3307 women are from the poor and middle-class sections. All the respondents of the study are not highly educated. 13.23 percent of women completed their studies to a higher level. A vast majority of women completed their studies up to the secondary and primary levels which are 38.79 percent and 32.31 percent each. About 15.67 percent of women from the study had no chance of an education. About half of the women from the study are currently working in different kinds of fields. A bulk amount of women are not pregnant at the time of the survey. Only 916 women were pregnant at this time although the rest of the 16106 women were not pregnant. A few women were breastfeeding their children and that was around 20.34 percent. The remainder of them which is 79.66 percent of women who were not breastfeeding at the time of the study. All of the women are ever-married so a greater number of them have children. The number of children of women is expressed by the variable children ever born which is categorized into 3 sections. The respondent who does not have any children are categorized as None and a few women which is 1447 are not having any child at the time. Some of them have one or two children which are combined as categories 1-2. The maximum number of women are fallen into this category. About 8369 women have less than or equal to two children. Women who have more than two children are categorized as 3 or more and about 7206 amount of women are fallen into this category. Some of the respondents gave birth to their children in the last five years. The factor of birth in the last 5 years indicates that approximately one-third of the women in the study did not give birth to any children. 37.64 percent of women gave birth to more than 2 children in the last five years period. Very least amount of women, about less than 1 percent gave birth to more than three children in the last five years. Among all respondents, about 22 percent of women are having partners who are illiterate. Many of the women have a partner who has completed their education up to the primary or secondary level. 32.37 percent of them have a partner who accomplished their primary level of education, whereas 29.12 percent of them completed their secondary level of education. Also, there are some women about 16.50 percent whose partner has fulfilled their higher education. Almost every respondent, about 98 percent in the study are having water from a safe source. A very small portion of people is collecting water from an unsafe source which is at most 2 percent. A vast majority of respondents are using a hygienic toilet. 12035 women from the total population have a hygienic toilet facility, meanwhile, only 4987 women do not get any hygienic toilet facility. Cooking fuel has two categories, improved and unimproved. Many of the respondents do not get to use improved fuel for cooking. Only 19.93 percent of women are using improved cooking fuel since 80.07 percent of women are using unimproved cooking fuel. These are all the factors that are chosen for the study to predict the malnutrition of women in Bangladesh. A nutritious diet is essential for leading a healthy lifestyle. The factors discussed in this section are closely related to a nutritious diet. Different types of female populations based on different factors like age, region, division, socioeconomic status, etc. have different types of nutritional situations. To predict the presence of malnutrition among these women, six ML-based classifiers has used, and present the performances of these models are in the following chapter. Based on the effectiveness of these models the best classifier will be chosen for the prediction of the nutritional status of women in Bangladesh.

**Table 2:** Background and demographic characteristics of the women in survey

Factor	Category	No. of observations	Percentage
Age (AG)	15-24(A1)	4311	25.32
	25-34(A2)	6126	35.99
	35-49(A3)	6585	38.69
	Total	17022	100
Residence ﻿(RE)	Rural(Ru)	10839	63.68
	Urban(Ur)	6183	36.32
	Total	17022	100
Division ﻿(DIV)	Barisal(Br)	1804	10.59
	Chattogram(Ch)	2359	13.86
	Dhaka(Dh)	2535	14.89
	Khulna(Kh)	2283	13.41
	Mymensingh(My)	1859	10.92
	Rajshahi(Rj)	2221	13.05
	Rangpur(Rn)	2151	12.64
	Sylhet(Sy)	1810	10.63
	Total	17022	100
	(Barisal,Chattagram,	8981	52.76
	Dhaka,Khulna)(Sou)		
	(Mymensingh,Rajshahi,	8041	47.24
	Rangpur,Sylhet)(Nor)		
Wealth Index (WI)	Poor(P)	6510	38.24
	Middle(M)	3307	19.43
	Rich(R)	7205	42.33
	Total	17022	100
Education ﻿(ED)	No education(NE)	2667	15.67
	Primary(Pri)	5500	32.31
	Secondary(Sec)	6603	38.79
	Higher(High)	2252	13.23
	Total	17022	100
Currently Working ﻿(CW)	No(N)	8609	50.58
	Yes(Y)	8413	49.42
	Total	17022	100
Currently Pregnant ﻿(CP)	No(N)	16106	94.62
	Yes(Y)	916	5.38
	Total	17022	100
Currently Breastfeeding ﻿(CB)	No(N)	13559	79.66
	Yes(Y)	3463	20.34
	Total	17022	100
Children Ever Born ﻿(CH)	None(No)	1447	8.50
	1-2($>=$2)	8369	49.17
	3 or more(<2)	7206	42.33
	Total	17022	100
Birth in last 5 years ﻿(B5)	None(No)	10566	62.07
	1-2($>=$2)	6407	37.64
	3 or more(<2)	49	0.29
	Total	17022	100
Partner Education ﻿(PE)	No education(NE)	3746	22.01
	Primary(Pri)	5510	32.37
	Secondary(Sec)	4957	29.12
	Higher(High)	2809	16.50
	Total	17022	100
Water Source ﻿(WS)	Safe(SF)	16663	97.89
	Unsafe(USF)	359	2.10
	Total	17022	100
Toilet Facility ﻿(TF)	Hygenic(HY)	12035	70.70
	Unhygenic(UHY)	4987	29.29
	Total	17022	100
Cooking Fuel ﻿(CF)	Improved(I)	3393	19.93
	Unimproved(UI)	13629	80.07
	Total	17022	100

The study is aimed at classifying the malnutrition of women in Bangladesh and finding a well-fitted machine-learning model for the classification. The study has selected fourteen different variables that may be related to the malnutrition problem. The machine-learning models and the chosen data set for the study are mentioned in the preceding chapters. This section will explore the performances of ML-based algorithms. First, the study tracked the significance of these variables using Chi-square at a 5 percent significance level and then it fitted the ML-based algorithms with the significant variables that are related to the nutritional status of women.

*Identification of risk factors of malnutrition using Chi-Square:* Several factors have been taken for predicting the malnutrition of women in Bangladesh based on the previous study as it is stated before. The significance of these variables is checked with the Chi-square $(\chi ^2)$ test. The Chi-square test was conducted with a significance level of 5 percent. The result is evaluated with the *p*-*value* of every variable.

Table 3 represents the Chi-square test of the variables with *p*-values and the expected frequencies of the categories. All the factors have large $(\chi ^2)$ values with *p*-value $<0.001$ except for the water source. The water source has $(\chi ^2)$ = 4.238 with *p*-value = 0.04, although the value of p is less than 0.05. So all the variables that are chosen for the study are significant under the significant level of 5 percent. So the study used these 14 variables as the risk factors for malnutrition of women in Bangladesh for BDHS, 2017-18, and predicted the malnutrition of women using six ML-based algorithms and evaluating their performances.

Table 3 also shows the number of people who are been affected by malnutrition based on the different variables. The data had been taken from BDHS, 2017-18 for this study. Considering the findings of the study, the women of age greater than 35 had malnutrition problems against the others. The women who are lived in rural areas very much suffer from the problem of malnutrition. The southern area which includes Barisal, Chattagram, Dhaka, and Khulna had 55 percent of malnourished women from the whole sample. The higher-educated women and women with higher-educated husbands had the least number of malnutrition cases than others. The poor women are malnourished which is being underweight and the rich women are suffering from malnutrition which is being overweight. The women who were having a child faced malnutrition against the women with no child. The women having unimproved cooking fuel are malnourished with compare to women having improved cooking fuel. There is minimal data on unsafe water sources and unhygienic toilet facilities but among them, there is a great portion of women who have malnutrition whether underweight or overweight.

**Table 3:** Association between factors and nutritional status among Observed ($O_i$) and Expected ($E_i$) for women from BDHS, 2017-18

Factors	Category	Normal	Malnutrition	Total	Chi-square (*p*-value)
		$O_i (E_i)$	$O_i (E_i)$		
Total		9331	7691	17022	
Age	15-24	2739(2363.2)	1572(1947.8)	4311	196.716 (<0.001)
	25-34	3301(3358.1)	2825(2767.9)	6126	
	35-49	3291(3609.7)	3294(2975.3)	6585	
Residence	Rural	6352(5941.6)	4487(4897.4)	10839	172.682 (<0.001)
	Urban	2979(3389.4)	3204(2793.6)	6183	
Division	North Area	4588(4407.9)	3453(3633.1)	8041	30.883 (<0.001)
	South Area	4743(4923.1)	4238(4057.9)	8981	
Education	No education	1566(1462.0)	1101(1205.0)	2667	63.908 (<0.001)
	Primary	3066(3015.0)	2434(2485.0)	5500	
	Secondary	3624(3619.6)	2979(2983.4)	6603	
	Higher	1075(1234.5)	1177(1017.5)	2252	
Currently Pregnant	No	8770(8828.9)	7336(7277.1)	16106	16.147 (<0.001)
	Yes	561(502.1)	355(413.9)	916	
Currently Working	No	4494(4719.2)	4115(3889.8)	8609	48.132 (<0.001)
	Yes	4837(4611.8)	3576(3801.2)	8413	
Currently Breastfeeding	No	7164(7432.7)	6395(6126.3)	13559	105.658 (<0.001)
	Yes	2167(1898.3)	1296(1564.7)	3463	
Wealth index	Poor	4025(3568.6)	2485(2941.4)	6510	327.544 (<0.001)
	Middle	1927(1812.8)	1380(1494.2)	3307	
	Rich	3379(3949.6)	3826(3255.4)	7205	
Children everborn	None	867(793.2)	580(653.8)	1447	16.606 (<0.001)
	1-2	4548(4587.7)	3821(3781.3)	8369	
	3 or more	3916(3950.1)	3290(3255.9)	7206	
Birth in last 5 years	None	5592(5792.0)	4974(4774.0)	10566	40.517 (<0.001)
	1-2	3709(3512.1)	2698(2894.9)	6407	
	3-4	30(26.9)	19(22.1)	49	
Partner’s Education	No education	2224(2053.5)	1522(1692.5)	3746	167.232 (<0.001)
	Primary	3188(3020.4)	2322(2489.6)	5510	
	Secondary	2659(2717.3)	2298(2239.7)	4957	
	Higher	1260(1539.8)	1549(1269.2)	2809	
Water source	Safe	9115(9134.2)	7548(7528.8)	16663	4.238 (0.04)
	Unsafe	216(196.8)	143(162.2)	359	
Toilet facility	Hygenic	6271(6597.3)	5764(5437.7)	12035	121.89 (<0.001)
	Unhygenic	3060(2733.7)	1927(2253.3)	4987	
Cooking fuel	Improved	1446(1860.)	1947(1533.0)	3393	254.666 (<0.001)
	Unimproved	7885(7471.0)	5744(6158.0)	13629	

*Performances of the machine learning methods:* Six distinct algorithms for machine learning were employed in this investigation to classify the women in the dataset as malnourished or nourished. Six different machine learning techniques are Naïve Bayes, two types of Decision Trees (Classification and Regression Tree and C5.0 Classification), Logistic Regression, Random Forest, and Gradient Boosting Machine. The dataset was partitioned into the training dataset and test dataset. Seventy-five percent of the data was taken as training data (training data set = 12766) and 25 percent was selected as test data (test data set = 4256). The performance of these methods is given below.

*Naïve Bayes:* The Naïve Bayes model is constructed by projecting probabilities of class labels ’No’ or ’Yes’ for malnutrition, which includes the chance that a given tuple belongs to a specific class. The prediction results with Naïve Bayes performance settings are shown in Table 4(a) for both training data and test data. Based on training data the confusion matrix shows that NB can predict 4890 people as normal, whereas 2683 people as malnourished. The accuracy of the training data is seen as 0.593 means that 59.3 percent time the model predicted correctly in relation to the total number of predictions made. On the other side, test data has an accuracy of 0.590. For the test data set with respect to the total number of predictions produced, the model forecasted accurately 59 percent of the time. The model has a sensitivity of 0.615 and 0.607 respectively for both training and test data. The model has the ability to predict correctly about 60 percent for both test and training data when the cases are genuinely positive known as normal. Also, the model can correctly predict negative cases 55.8 percent and 56.1 percent individually for training and test data when the cases are negative also known as malnutrition existence. The PPV and NPV of NB for training data are 0.697 and 0.467 and for test data are 0.696 and 0.463. The $F_1$ score of NB determines that 65.3 and 64.8 percent of the time the model is collect positive normal cases while remaining accurate with the examples it does catch throughout the entire training and test data set, which is deemed satisfactory. Cohen’s Kappa values for NB are estimated to be 0.166 and 0.162 for both segmented data sets, indicating “slight” discriminative power.

**Fig. 3:**
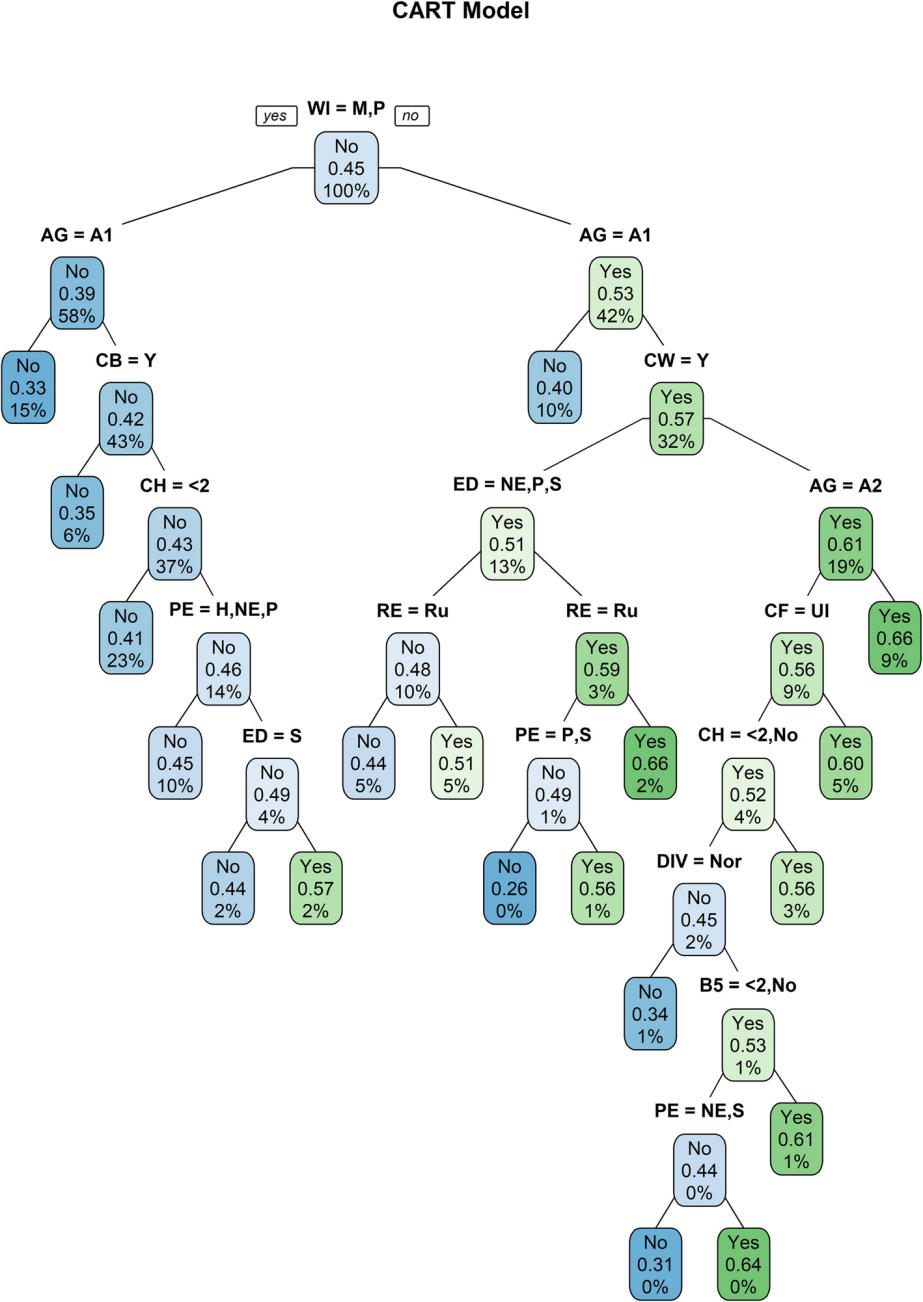
CART plot for predicting malnutrition of women in Bangladesh using BDHS, 2017-18 data

*Classification and Regression Tree:* The Classification and regression tree is a type of decision tree employed in order to predict women’s nutritional status. The model is fitted using the training data set with the complexity parameter (CP) 0.001. Figure 3 displayed the decision tree for the measured variable. The tree starts with the node using the WI factor. Then by using the Gini index, the factors are split. Branches are grown from the initial node for each outcome of the Gini index. Using this tree the prediction had been made for the training data and test data and evaluate its performance.

Table 4(b) displays the prediction result using Classification and Regression Tree effectiveness levels both for the test and training data. The confusion matrix demonstrates that CART can classify 5622 people as healthy and 2107 persons as malnourished based on training data as well as 1814 as healthy and 672 as malnutrition. The accuracy of the training data is 0.605, which indicates that the model successfully predicted 60.5 percent of the time with respect to the total number of projections produced. Test data, on the opposite side, has an accuracy of 0.591. In terms of the total number of estimates provided for the test data set, the model can forecast correctly 59.1 percent of the time. The model has sensitivity values of 0.607 and 0.592 both to training and test data, accordingly. When the cases are actually normal, the model can properly predict roughly 60 percent of the time for both test and training data. Furthermore, the specificity of the CART algorithm is 0.602 and 0.587 for both training and test data sets and it interprets that when the examples are negative or have malnutrition problems, the machine can properly anticipate them 60.2 percent of the time for training data and 58.7 percent of the time for test data. The PPV and NPV of CART for training data are 0.801 and 0.366, respectively, whereas for test data they are 0.796 and 0.346. PPV indicates that about 80 proportion of women were actually normal to all those who received positive normal test results in both training and test data sets. NPV stipulates that the probability that a woman with a negative test result does have a malnutrition problem is close to 35 percent. The $F_1$ score of CART shows that the model’s ability to capture positive normal cases while remaining accurate with the examples it does capture is 69.1 and 67.9 times out of 100 over the whole training and test data sets, respectively. For both segmentation, Cohen’s Kappa coefficients for CART are assessed to be 0.174 and 0.147, indicating “slight” exclusionary potential.

**Fig. 4:**
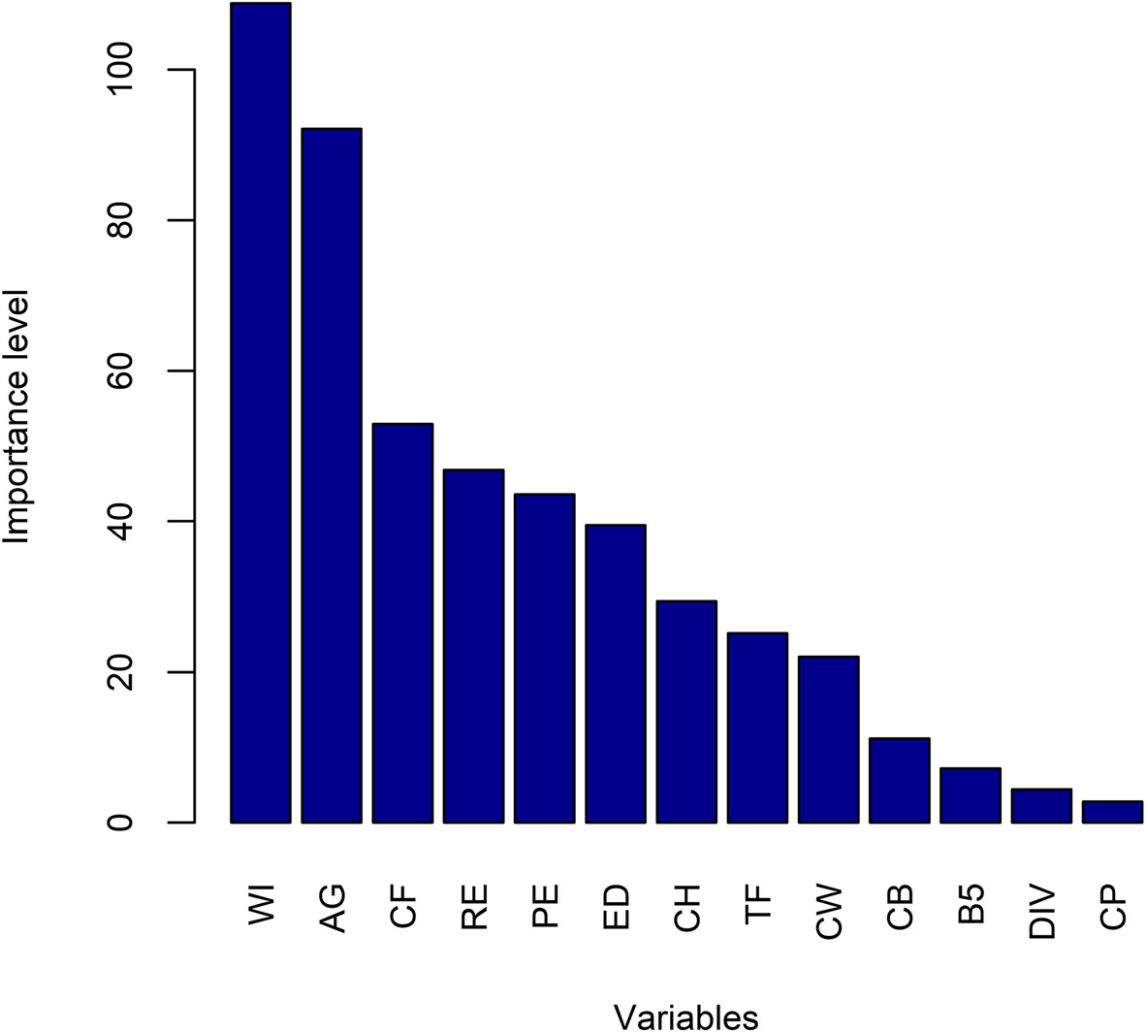
Importance level of factors for the nutritional status of women for BDHS, 2017-18 using CART

Also, the CART model shows the importance of the variables for this classification in Fig. 4. CART demonstrates that variables WI and AG are the most influential variables for nutritional status. CF, RE, PE, ED, CH, TF, and CW are the variables with mid-type of importance. The four variables that have the least importance for the status of malnourishment are CB, B5, DIV, and CP. WS has no influence on being malnourished or not.

*C5.0 Classification:* Another decision tree is constructed to envisage the malnutritional status of women in Bangladesh from the (BDHS) 2017-18 data set. This decision tree is known as C5.0 Classification.

**Fig. 5:**
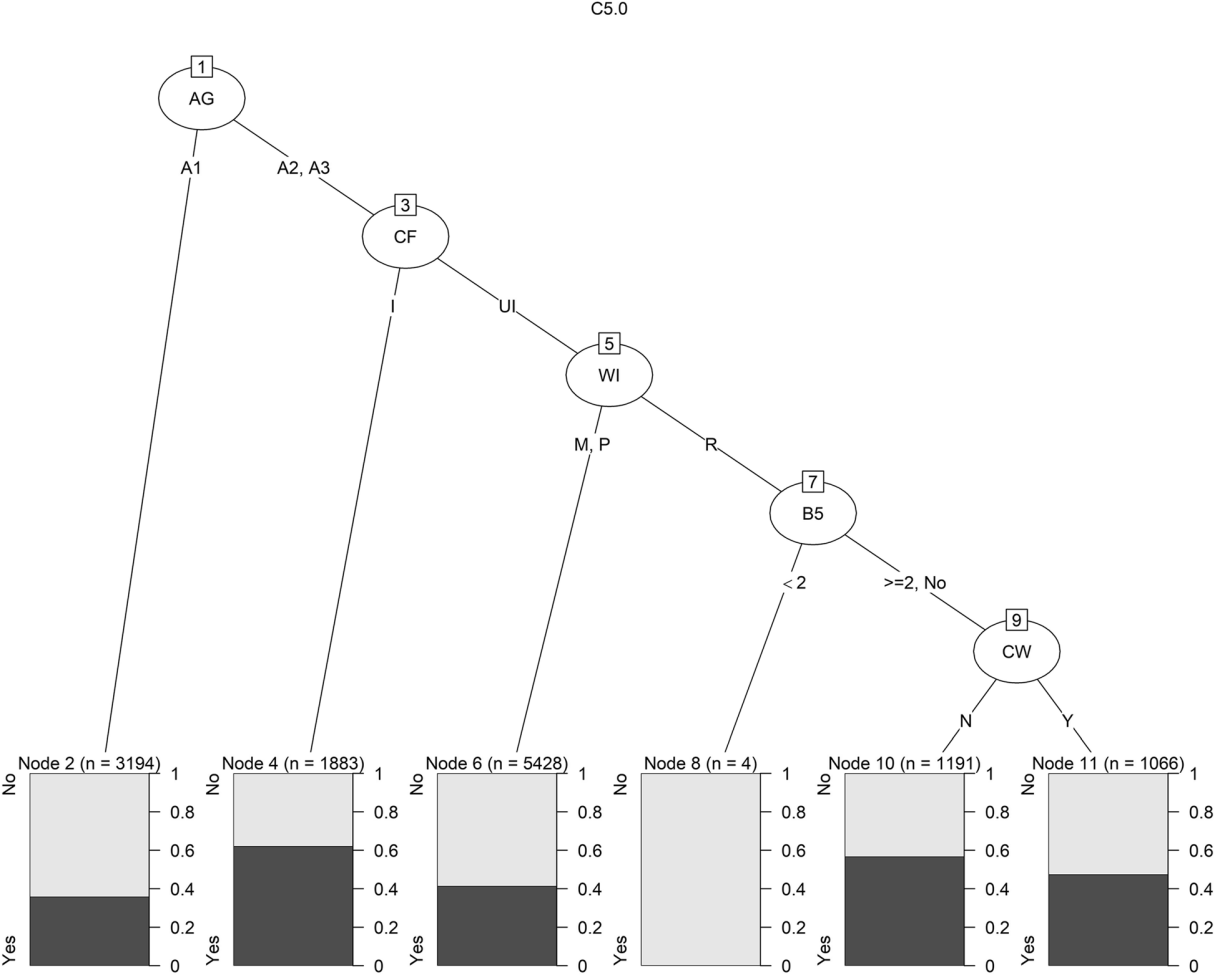
C5.0 plot for predicting Malnutrition of women in Bangladesh using BDHS, 2017-18 data

This tree is generated using minimum cases of 900 where it has to be included in at least two of the splits. The tree is formed using five variables AG, CF, WI, B5, and CW. The AG variable has the highest information gain, so it is chosen as the first splitting attribute. Then the following variables are selected for splitting by their information gain. This tree in Fig. 5 is used to estimate the number of cases of malnutrition.

Table 4(c) shows the predictive performance for both the test and training data using C5.0 Classification efficacy levels. According to the contingency table, C5.0 can categorize 5789 as normal women and 1846 as malnourished based on training data, along with 1904 as normal and 611 as malnutrition. The trained data accuracy is 0.598, meaning the model correctly forecasted around 60 percent of the time according to the entire amount of forecasts generated. On the other hand, test data has an accuracy of 0.591. The model can forecast properly 59.1 percent of the time based on the total number of estimations supplied for the test data set. The model exhibits sensitivity values of 0.597 and 0.589 to training and test data, respectively. When the cases are genuinely affirmative to normal, the model can correctly predict around 60 percent of the time for both test and training data. Furthermore, the specificity of the C5.0 method is 0.601 and 0.598 for both training and test data sets, implying that when the instances are negative to normal, the machine can correctly predict them 60 percent of the time for training data and test data independently. C5.0’s PPV and NPV for training examples are 0.825 and 0.321, correspondingly, while for test cases they are 0.823 and 0.315. In all training and test data sets, PPV reveals that approximately 82 percent of women were normal compared to all those who had negative malnutrition test findings. According to NPV, the likelihood that a woman with a negative test result for normal does have a problem with malnutrition is close to 32 percent. C5.0’s $F_1$ score indicates that the model correctly predicts 69.3 times out of 100 over the whole training data set. For the test data set $F_1$ score is close to the score of training data which is 0.686. This value is considered an ’Ok’ score. Cohen’s Kappa coefficients for C5.0 are 0.153 and 0.143 for both segments, demonstrating “slight” discriminatory potential.

*Logistic Regression:* Logistic Regression is fitted with the reference category ’No’ for the training data to predict malnutrition among the women. The prediction performance for both the test and training data is shown in Table 4(d) using Logistic Regression. LR can classify 6729 women as normal and 655 as malnutrition for training data with an accuracy of 0.578. The LR can correctly envisage 57.8 percent over the entire training data. For the test data set the accuracy of LR is 0.594 where it can classify 1799 as normal and the opposite 727 as malnourished. The model includes sensitivity and specificity of 0.569 and 0.695 for the training data. It can correctly predict about 57 percent when the cases are affirmative to normal and can predict correctly about 70 percent when the cases are malnourished. On the other side for test data, the correctly predictive normal cases are 59.7 percent and 58.5 percent for the cases of correctly predictive malnourished cases. The PPV of training data for LR indicates that 96 percent of individuals are normal in comparison with normal women in test findings, but for the test data it is a bit low compared with training data which is 77.7 percent. A woman with malnutrition positive on the test does have a problem only 11.4 percent for the training data set which is expressed by NPV and for the test data set, it is 37.4 percent. $F_1$ of LR reveals that the capacity of the model to capture positive normal cases while remaining accurate with the examples it does capture is 71.4 percent and 67.5 percent for training and test data each, which is considered an acceptable figure. The Kappa of training data is very poor for this model. It is only 0.079 which indicates a “slight” discriminative power. The test data has a 0.143 Kappa coefficient which also indicates the same discriminative potential.

**Fig. 6:**
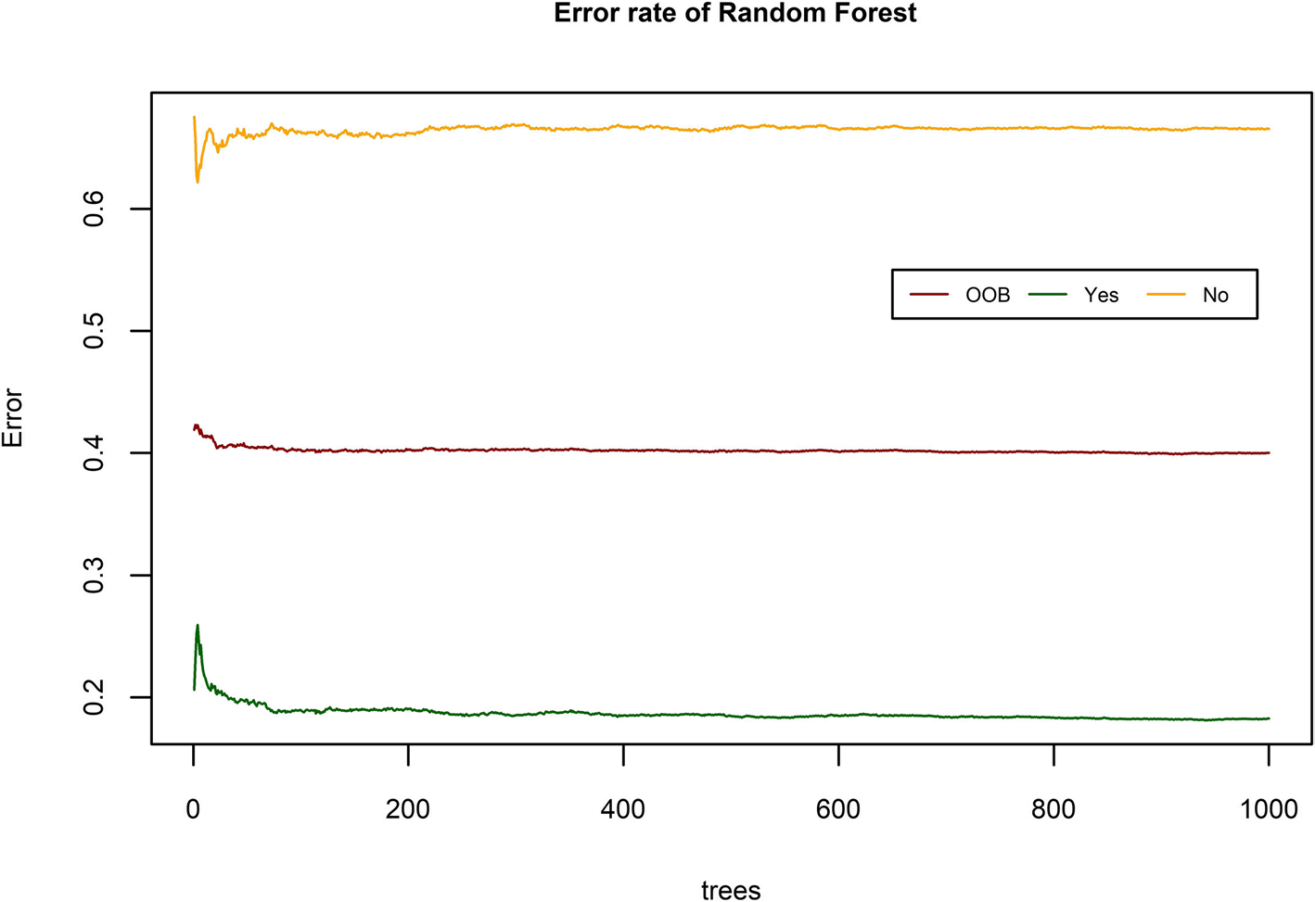
Error rate of Random Forest against the number of trees

*Random Forest:* Random Forest is a bagging technique that is used in this study to fit the model for predicting the nutritional status of women in Bangladesh. 1000 decision trees are used with randomly chosen two variables for constructing a tree. Two variables for the construction hold a minimum “Out of Bag” (OOB) error which is 40 percent. Figure 6 has shown the error rate against the number of trees for RF. The error rate is constant as the number of trees increased. So 1000 trees are enough to produce a good prediction of malnutrition with the test data and training data for women.

Table 4(e) displays the prediction performance for both the test and training data using Random Forest. RF can predict with the same accuracy for both training and test data. It can classify 5735 women and 1902 women as normal for both training and test data separately together with 1925 and 658 women as malnourished. The model can correctly identify 60 percent of the time according to the training and test data. All the assessment parameters of RF are nearly same for the both data set. It has a sensitivity and specificity of about 60 percent for both data sets. Around 82 percent of women are actually normal to those who receive positive normal test results and around 34 percent with malnutrition who are tested as malnourished according to PPV and NPV of both training and test data. The $F_1$ score of RF suggested that the model has the capacity to capture positive normal cases while remaining accurate with the cases it does capture is 0.692, which is considered to be an acceptable number. The Kappa coefficients are 0.159 and 0.167 for both of the data indicating a “slight” agreement.

*Gradient Boosting Machine:* A gradient Boosting Machine has been fitted for the status of nutrition for women in Bangladesh. A total of 2000 decision trees are used to fit the GBM with a shrinkage of 0.01. The assessment values of the Gradient Boosting Machine are displayed in Table 4(f). The accuracy of 60 percent by correctly predicting 5442 women as normal and 2232 as malnourished for the training data set. The test data has the same accuracy where it categorized 1796 individuals for normal and 739 for malnutrition correctly. GBM can predict 60 percent of the time correctly as normal when the test result is normal for the woman for both data as stated by sensitivity. Both data have the correct prediction of 59 percent with malnutrition when the actual test result is malnutrition as per specificity. PPV and NPV are identical for both parts of the data. GBM has a PPV of 78 percent and an NPV of 38 percent. GBM’s $F_1$ scores reveal that the model’s capacity to capture positive normal cases while remaining accurate with the examples it does capture is 0.68, which is considered as ’Ok’ for both partitions. It has “slight” agreement in the opinion of the Kappa coefficient with results of 0.169 and 0.161 for both data sets.

**Fig. 7:**
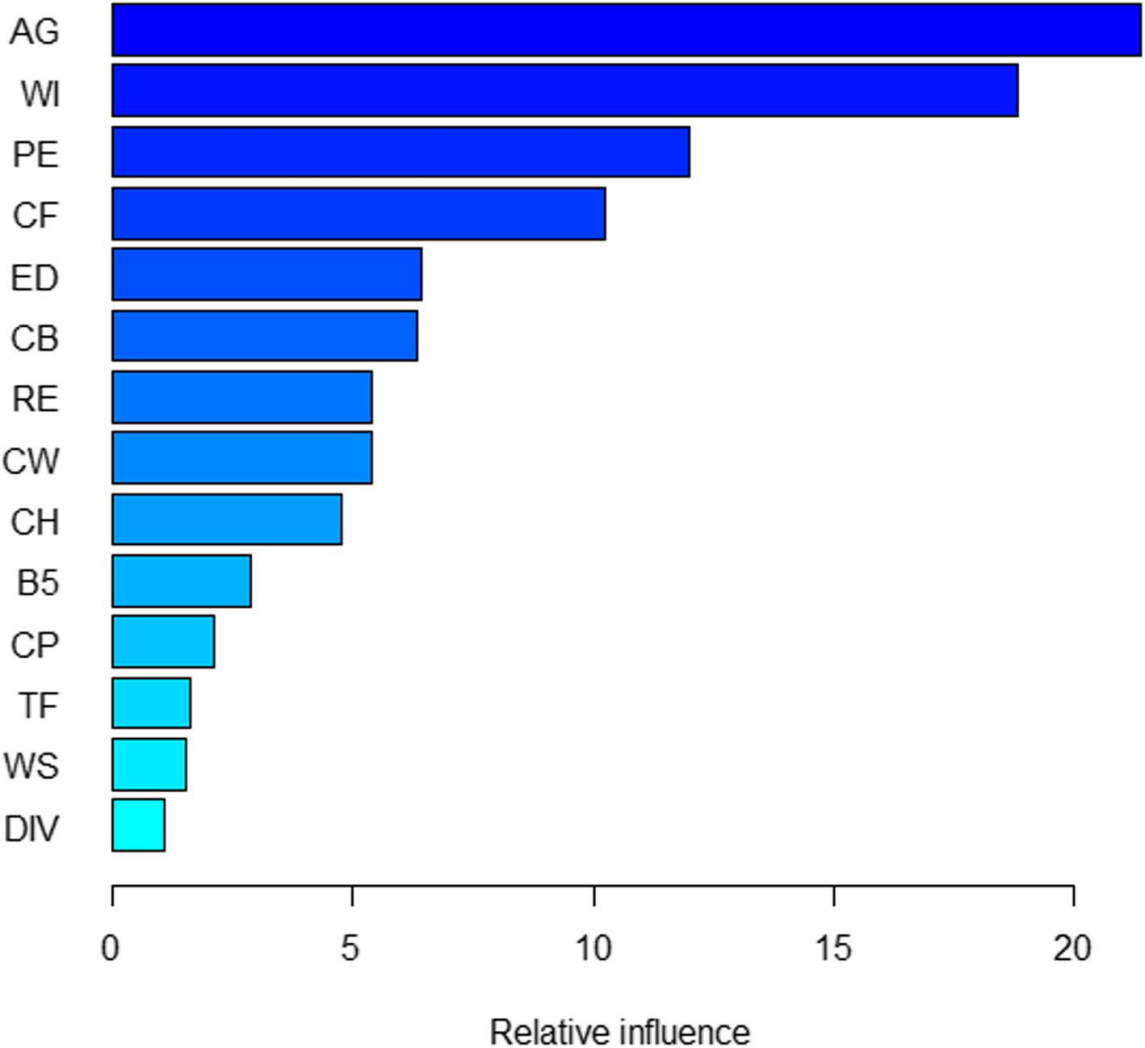
Factors influence the nutritional status of women for BDHS, 2017-18 using GBM

In addition, the GBM model in Fig. 7 indicates the relative influence of the factors for this categorization. According to GBM, the variables AG and WI have the greatest influence on malnutrition. The factors having medium relevance include PE, CF, ED, CB, RE, CW, CH, and B5. CP, TF, DIV, and WS are the four factors with minimal effect on the nutritional status of women in Bangladesh.

**Table 4:** The performance metrics of ML-based algorithms with their confidence interval for predicting malnutrition of women in Bangladesh using BDHS, 2017-18 data

	Training dataset			Test dataset		
**(a) Naïve Bayes**						
Confusion Matrix	Category	No	Yes	Category	No	Yes
	No	4890	2127	No	1610	704
	Yes	3066	2683	Yes	1042	900
Sensitivity	0.615 (0.604, 0.626)			0.607 (0.588, 0.626)		
Specificity	0.558 (0.544, 0.572)			0.561 (0.537, 0.585)		
Positive Predictive Value	0.697 (0.686, 0.708)			0.696 (0.677, 0.715)		
Negative Predictive Value	0.467 (0.454, 0.480)			0.463 (0.441, 0.485)		
Accuracy	0.593 (0.584, 0.602)			0.590 (0.575, 0.605)		
$F_1$Score	0.653			0.648		
Kappa	0.166			0.162		
**(b) CART**						
Confusion Matrix	Category	No	Yes	Category	No	Yes
	No	5622	1395	No	1841	473
	Yes	3642	2107	Yes	1270	672
Sensitivity	0.607 (0.597, 0.617)			0.592 (0.575, 0.609)		
Specificity	0.602 (0.586, 0.618)			0.587 (0.558, 0.616)		
Positive Predictive Value	0.801 (0.792, 0.810)			0.796 (0.780, 0.812)		
Negative Predictive Value	0.366 (0.354, 0.378)			0.346 (0.325, 0.367)		
Accuracy	0.605 (0.597, 0.613)			0.591 (0.575, 0.605)		
$F_1$Score	0.691			0.679		
Kappa	0.174			0.147		
**(c) C5.0**						
Confusion Matrix	Category	No	Yes	Category	No	Yes
	No	5789	1228	No	1904	410
	Yes	3903	1846	Yes	1331	611
Sensitivity	0.597 (0.587, 0.607)			0.589 (0.572, 0.606)		
Specificity	0.601 (0.584, 0.618)			0.598 (0.568, 0.628)		
Positive Predictive Value	0.825 (0.816, 0.834)			0.823 (0.807, 0.839)		
Negative Predictive Value	0.321 (0.309, 0.333)			0.315 (0.294, 0.336)		
Accuracy	0.598 (0.589, 0.607)			0.591 (0.576, 0.606)		
$F_1$Score	0.693			0.686		
Kappa	0.153			0.143		
**(d) Logistic Regression**						
Confusion Matrix	Category	No	Yes	Category	No	Yes
	No	6729	288	No	1799	515
	Yes	5094	655	Yes	1215	727
Sensitivity	0.569 (0.560, 0.578)			0.597 (0.579, 0.615)		
Specificity	0.695 (0.666, 0.724)			0.585 (0.558, 0.612)		
Positive Predictive Value	0.959 (0.954, 0.964)			0.777 (0.760, 0.794)		
Negative Predictive Value	0.114 (0.106, 0.122)			0.374 (0.352, 0.396)		
Accuracy	0.578 (0.569, 0.587)			0.594 (0.579, 0.609)		
$F_1$Score	0.714			0.675		
Kappa	0.079			0.153		
**(e) Random Forest**						
Confusion Matrix	Category	No	Yes	Category	No	Yes
	No	5735	1282	No	1902	412
	Yes	3824	1925	Yes	1284	658
Sensitivity	0.600 (0.590, 0.610)			0.597 (0.580, 0.614)		
Specificity	0.600 (0.583, 0.617)			0.615 (0.586, 0.644)		
Positive Predictive Value	0.817 (0.808, 0.826)			0.822 (0.806, 0.838)		
Negative Predictive Value	0.335 (0.323, 0.347)			0.339 (0.318, 0.360)		
Accuracy	0.600 (0.592, 0.608)			0.602 (0.587, 0.617)		
$F_1$Score	0.692			0.692		
Kappa	0.159			0.167		
**(f) Gradient Boosting**						
Confusion Matrix	Category	No	Yes	Category	No	Yes
	No	5442	1575	No	1796	518
	Yes	3517	2232	Yes	1203	739
Sensitivity	0.607 (0.597, 0.617)			0.599 (0.581, 0.617)		
Specificity	0.586 (0.570, 0.602)			0.588 (0.561, 0.615)		
Positive Predictive Value	0.776 (0.766, 0.786)			0.776 (0.759, 0.793)		
Negative Predictive Value	0.388 (0.375, 0.401)			0.381 (0.359, 0.403)		
Accuracy	0.601 (0.593, 0.609)			0.596 (0.581, 0.611)		
$F_1$Score	0.681			0.676		
Kappa	0.169			0.161		

*Evaluation of the efficacy of machine learning methods:* The six ML algorithms NB, CART, C5.0, LR, RF, and GBM are applied for predicting the malnutrition of women from BDHS, 2017-18. The algorithms are fitted with 75 percent fixed data as training from the overall data set. But the result can not be evaluated with one training data set because it would arise biases and increase variability. So this study has taken six protocols for checking the performance of these ML algorithms.

Six protocols that are taken are 2, 3, 5, 7, 10, and 11. All six models run under these protocols and measured the assessment values which are presented in Table 5. The models have very few changes in their performance from the k-folds. Naïve Bayes has an accuracy of around 59 percent for every k-fold. As increases the fold, the accuracy of NB slightly getting better. The Kappa value is better for 7-fold validation. It has a value of 0.158 which indicates “slight” agreement. $F_1$ score is almost the same for all the six protocols of NB which are 67 percent. 7-fold has the better result for SE, SP, PPV, and NPV. The next algorithm is CART which has a considerably better result than NB. CART has an accuracy of around 60 percent for all six protocols. Kappa is a little much worse than NB which is 0.149 for 3-fold and 10-fold. CART has a $F_1$ score close to 69 percent. CART can predict correctly about 80 percent for every k-fold data when the cases are positive for normal. It has the highest SP of 0.346 for 10-fold validation. PPV and NPV are nearly the same for every fold of CART. Another decision tree C5.0 classification has almost the same accuracy as CART. But it has a better Kappa score which is 0.168 the highest of six folds. The $F_1$ score is 69 percent for 2-fold and 11-fold and the remaining have 68 percent of $F_1$ value. It has SE at around 80 percent, SP at around 34 to 39 percent, and PPV and NPV at almost 60 percent. C5.0 has the better result for 3rd fold as a comparison to others. Binary logistic regression is fitted for the model with cross-validation and it has the almost same result as the decision tree. But it has a better Cohen’s Kappa coefficient for every k-fold comparison to others before. The $F_1$ score is not as convenient as others. It has the second lowest $F_1$ values for every protocol. The sensitivity is 77 percent and the specificity is 39 percent for all. PPV and NPV are around 60 and 59 percent, respectively. The bagging technique also known as RF has 60 percent accuracy for the six protocols. It has low Kappa coefficients but has better $F_1$ scores among all. Nearly 70 percent of the time captures normal cases while remaining accurate with the example for every k-fold validation. It has also the highest sensitivity rate for the protocols. The specificity is around 33 percent and the PPV and NPV are almost 60 percent. With an accuracy of 60 percent to capture the nutritional status of women, GBM has the highest Kappa coefficients here. It has the second highest $F_1$ scores which are almost 69 percent. It has a total 80 percent of SE along with at most 37 percent SP for every fold. The PPV and NPV are as usual same as other models.

**Fig. 8:**
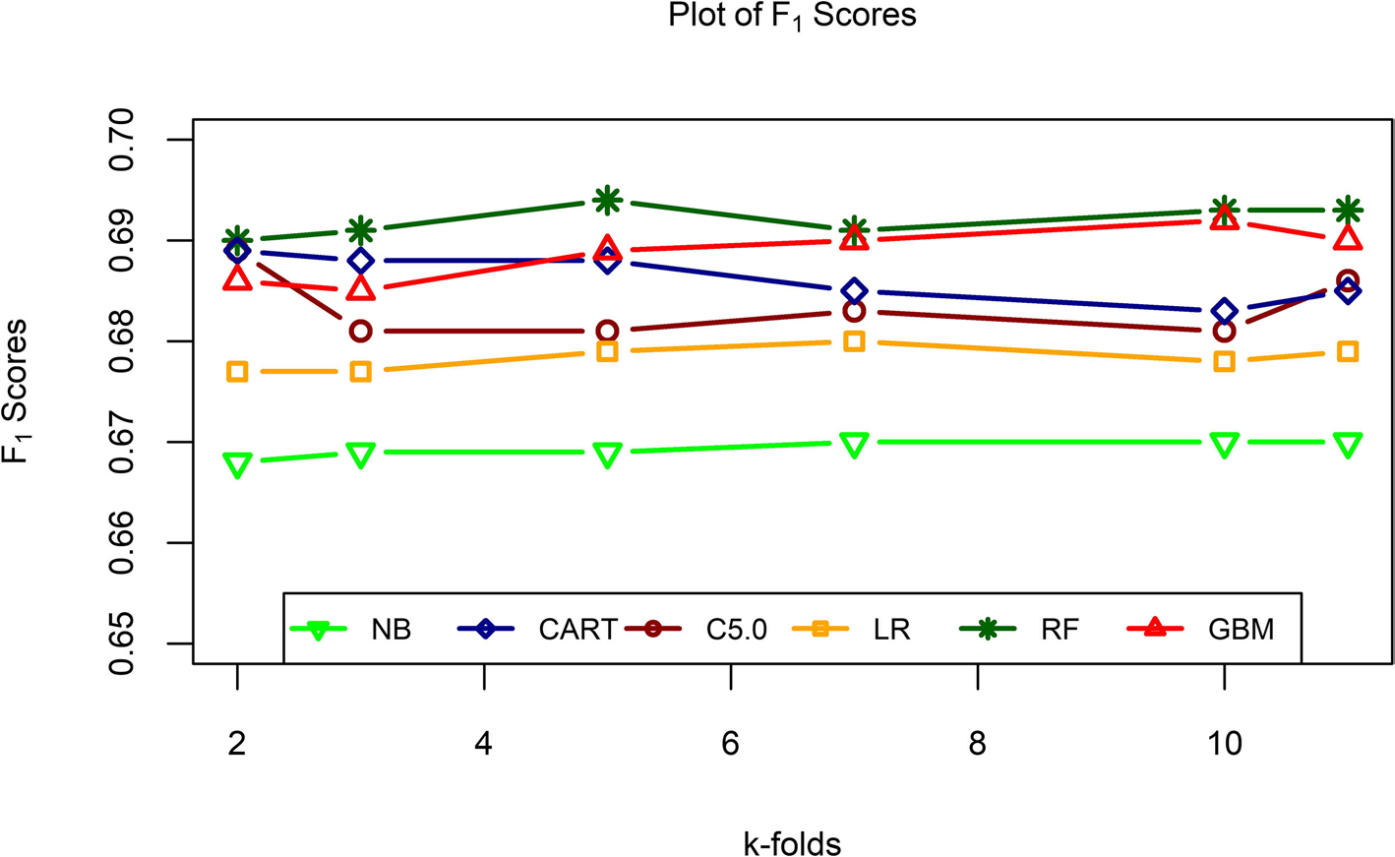
$F_1$ scores of the different ML algorithms across six protocols for malnutrition of women

All the models have the nearly same value of evaluation parameters. This study has chosen the $F_1$ score to try to evaluate the performance of the models. All the models have $F_1$ scores from 66 to 70 percent, roughly. That indicates that all the models are correct to collect both positive and negative cases which is recall while also being accurate with the cases it does capture which is precision. Figure 8 presented the $F_1$ scores of all models in their k-folds. The figure shows that RF has the highest score of $F_1$ among others. GBM has the second highest value of $F_1$ by increasing the k-fold values. The CART model has a greater value in 2-fold validation but as the k-fold increases, the values are decreasing for CART. Another DT, C5.0 has the same nature as CART as it is also decreasing by increasing the k-fold. LR and NB have the least $F_1$ values and among them, NB has the worst $F_1$ scores.

**Table 5:** Compare the performance of machine learning algorithms across six protocols for malnutrition of women

k	Ac	Kappa	$F_1$	SE	SP	PPV	NPV
**Naïve Bayes**							
2	0.590	0.151	0.668	0.751	0.395	0.601	0.568
3	0.592	0.154	0.669	0.754	0.397	0.602	0.571
5	0.593	0.157	0.669	0.751	0.401	0.604	0.572
7	0.594	0.158	0.670	0.753	0.401	0.604	0.573
10	0.593	0.156	0.670	0.753	0.399	0.603	0.571
11	0.593	0.156	0.670	0.752	0.400	0.603	0.571
**Classification and Regression Tree**							
2	0.594	0.146	0.689	0.821	0.319	0.594	0.596
3	0.595	0.149	0.688	0.814	0.329	0.595	0.593
5	0.594	0.147	0.688	0.814	0.328	0.595	0.592
7	0.593	0.147	0.685	0.806	0.335	0.595	0.588
10	0.594	0.149	0.683	0.798	0.346	0.597	0.585
11	0.593	0.147	0.685	0.806	0.335	0.595	0.588
**C5.0**							
2	0.599	0.159	0.689	0.809	0.344	0.599	0.598
3	0.601	0.168	0.681	0.778	0.386	0.606	0.589
5	0.597	0.160	0.681	0.785	0.369	0.602	0.587
7	0.599	0.164	0.683	0.787	0.371	0.603	0.590
10	0.599	0.164	0.681	0.781	0.378	0.604	0.588
11	0.600	0.163	0.686	0.797	0.360	0.602	0.594
**Logistic Regression**							
2	0.598	0.164	0.677	0.769	0.391	0.605	0.582
3	0.597	0.162	0.677	0.769	0.389	0.604	0.581
5	0.599	0.166	0.679	0.773	0.388	0.605	0.585
7	0.600	0.168	0.680	0.774	0.389	0.606	0.586
10	0.598	0.163	0.678	0.771	0.387	0.604	0.583
11	0.599	0.166	0.679	0.774	0.387	0.605	0.585
**Random Forest**							
2	0.597	0.153	0.690	0.817	0.330	0.597	0.598
3	0.596	0.149	0.691	0.826	0.317	0.595	0.600
5	0.599	0.157	0.694	0.827	0.323	0.597	0.606
7	0.599	0.157	0.691	0.820	0.332	0.598	0.602
10	0.600	0.157	0.693	0.826	0.325	0.597	0.606
11	0.599	0.156	0.693	0.824	0.326	0.597	0.604
**Gradient Boosting Machine**							
2	0.602	0.168	0.686	0.793	0.369	0.604	0.596
3	0.600	0.165	0.685	0.794	0.365	0.603	0.594
5	0.603	0.169	0.689	0.803	0.360	0.603	0.601
7	0.603	0.168	0.690	0.807	0.355	0.603	0.603
10	0.604	0.171	0.692	0.811	0.354	0.603	0.607
11	0.602	0.166	0.690	0.809	0.351	0.602	0.602

So, from Fig. 8, it is clearly shown that for this study RF can capture cases accurately for the data from BDHS, 2017-18 for women malnutrition.

**Fig. 9:**
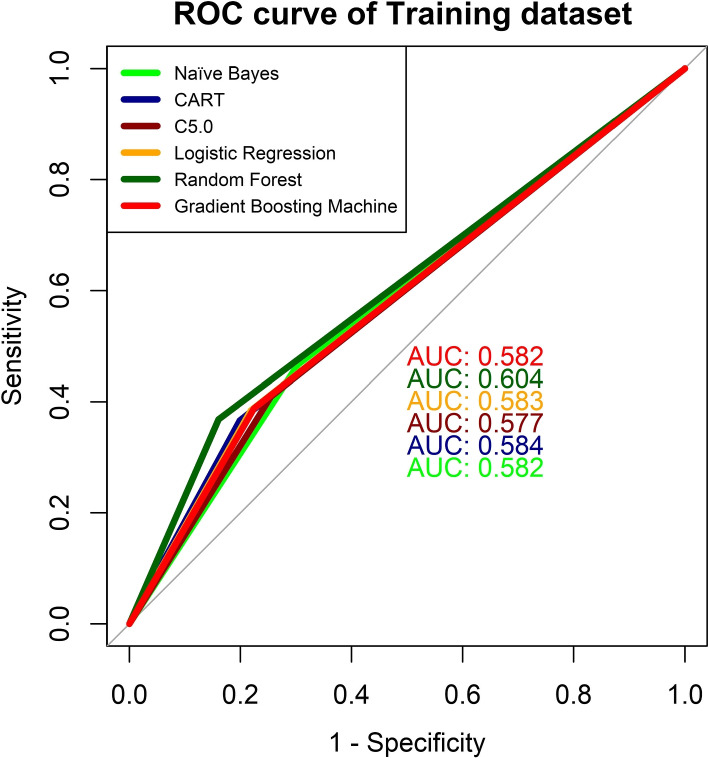
ROC curve of six machine learning algorithms for malnutrition of women using training dataset

**Fig. 10:**
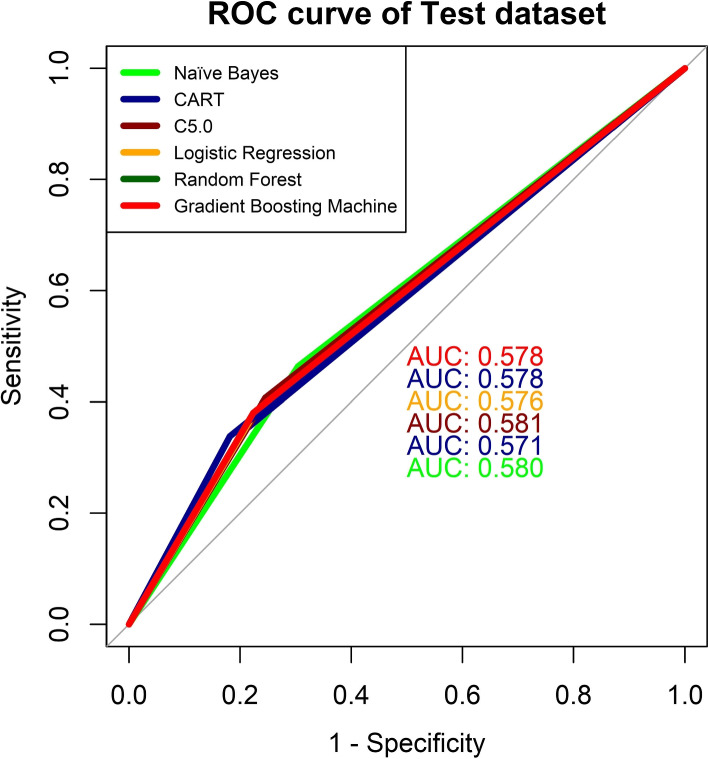
ROC curve of six machine learning algorithms for malnutrition of women using test dataset

The receiver operating characteristic system also known as the ROC curve has been produced for verifying the suggested systems. The ROC curves of six ML-based classifiers predicting women’s malnutrition in training and test data sets are shown in Figs. 9 and 10, respectively. From the figure of the ROC curve for training data, the Random Forest classifier has the highest AUC of 0.604 and the C5.0 classifier has the lowest AUC of 0.577. For the test data, it is totally reversible as the highest AUC is for the C5.0 classification which is 0.581. RF is also close to the C5.0 for the value of AUC. As a result, two different ROC curves showed two different models as better for prediction. So it is hard to tell the best predictive model for predicting the nutritional status of the female population with the ROC curve.

The performance of the models in training and test data does not differ from the performance in different protocols. So, based on the study of training and test data, the rank of these six models has been made according to the value of accuracy, Kappa, and $F_1$ score that is presented in Table 6.

**Table 6:** Rank of different models based on their accuracy, Cohen’s Kappa, and$F_1$score with rank value for training and test data

Model	Accuracy		Kappa		$F_1$		Rank total
	Training	Test	Training	Test	Training	Test	(Rank)
NB	0.593 (5)	0.590 (5)	0.166 (3)	0.162 (2)	0.653 (6)	0.648 (6)	27 (6)
CART	0.605 (1)	0.591 (4)	0.174 (1)	0.147 (5)	0.691 (4)	0.679 (3)	18 (2)
C5.0	0.598 (4)	0.591 (4)	0.153 (5)	0.143 (6)	0.693 (2)	0.686 (2)	23 (4)
LR	0.578 (6)	0.594 (3)	0.079 (6)	0.153 (4)	0.714 (1)	0.675 (5)	25 (5)
RF	0.600 (3)	0.602 (1)	0.159 (4)	0.167 (1)	0.692 (3)	0.692 (1)	13 (1)
GBM	0.601 (2)	0.596 (2)	0.169 (2)	0.161 (3)	0.681 (5)	0.676 (4)	18 (2)

The rank has been given for every individual performance of the models. Based on training data set accuracy, the CART model has the highest accuracy and has been ranked as first. GBM has the second-highest accuracy for the training data and the bagging method has been ranked third. LR has minimal accuracy for the training data. On the other hand, test accuracy has a different ranking position for the models. RF is the highest in accuracy for predicting malnutrition in women. The GBM model has the same rank as the training data. The decision trees are having the same accuracy(s) for the test data. All the Kappa values have ’Sligth’ in agreement but there are some differences. CART has the highest Kappa value for training data whereas the RF has the highest Kappa value for test data. For both training and test data, NB is in the last position based on the $F_1$ score. The LR is in the first position based on $F_1$ for training data and RF has the highest score of $F_1$ for test data. The sum of the ranks portrays that RF has the minimum sum of rank. CART and GBM have the same total rank. Another DT, C5.0 has the sum of rank 23. LR and NB are in the last for the total sum of rank, respectively.

The six machine learning algorithms are applied in the BDHS, 2017-18 data set to predict the nutritional status of women. The models have performed differently for training and test data sets. AS CART and GBM show the relative influences of the factors causing malnutrition. These models indicate that AG and WI have the maximum influence on malnutrition. Table 3 also indicates that as age increases the causing malnutrition also being increased and both AG and WI also have large Chi-square value that indicates a strong association with malnutrition. CF, PE, and RE are also strongly associated with malnutrition based on both CART, GBM, and Chi-square values. All the other factors have moderate influence and WS has the least influence on malnutrition among them. The performance of the models is evaluated with different assessment parameters. RF has the highest $F_1$ score in the cross-validation presented in Fig. 8. Also, the area under the curve (AUC) for RF and C5.0 is highest for training and test data, separately which is displayed in Figs. 9 and 10. RF has the highest rank according to the different parameters of assessment for both training and test data (Table 6). All the models that are used are fitted well for the data on women’s malnourishment and produce results that differ from each other. RF has outperformed other ML models as RF generates results based on a group of trees. Also, they take into account many decision trees that have less impact on particular data points, they are more resistant to extremes and noisy data than other algorithms, and also efficient. GBM also generates groups of trees and it also fits well in some aspects. However, according to the 3 performance metrics on training and test data, RF stands at the top than others. So,on the basis of all results, this study chooses RF as the best predictive machine learning classifier to predict the malnutrition of women.

The six algorithms based on machine learning are used to estimate women’s nutritional status. The classifiers responded differently on training and test data sets and gave different knowledge about the models. The chosen factors all are related to malnutrition and among them, AG and WI are the most associated factors. This research work has found that all the models can fit well with the data of women used in this study and among them RF can produce more vulnerable results about the prediction of malnutrition than others.

## Conclusion

A nutritious diet is essential for healthy living. Malnutrition is a serious condition caused by a lack of all nutrients and energy that the body requires. It is a major public health issue in all developing countries. It also remains a severe problem in Bangladesh, especially among women. Undernourished and overnourished both have been considered malnutrition and these are linked to a variety of infectious disorders. It also has negative repercussions for children, such as premature birth, decreased infection resistance, and an increased risk of death. It is critical to detect malnutritional causes early in order to prevent women from diseases that are aggravated by those factors. This project includes a detailed investigation for the identification and forecasting of malnourished women using ML classifiers. About seventeen thousand women are selected for conducting the research work. This study has selected some factors for detecting the nutritional status of women and finds that all the selected factors are significantly associated with the exposure variable. Among these variables, Age and Wealth Index are the most influential factors for the nutritional status of women and Water Source has the minimum influence on the malnourished problem. Age is an important factor as age increased the nutritional imbalance also increased because of negligence about the nutritional demand of the body based on age. Also, the socioeconomic status of a family is a predominant factor for malnutrition. These significant variables are also used as explanatory variables in ML-based algorithms for forecasting the malnourishment of women in Bangladesh.

In this study, six machine learning algorithms which are Naïve Bayes, Classification and Regression Tree, C5.0, Logistic Regression, Random Forest, and Gradient Boosting Machine are investigated for predicting if a female is malnourished based on risk indicators. The outcome of the investigation shows that for training and test data sets, Random Forest is the highest-ranked classifier among all based on accuracy, Kappa, and $F_1$ score. Random Forest has an accuracy of around sixty percent. Other models like Classification and Regression Tree and Gradient Boosting Machine also have an accuracy of sixty percent but by the performance of all other evaluating parameters such as Kappa and $F_1$, the Random Forest has a better result. Naïve Bayes, Logistic Regression, and C5.0 have an accuracy of less than sixty percent for the test data set of the research. Depending upon those assessing criteria, Random Forest has been ranked first. Also from the k-fold validation, Random Forest has the better $F_1$ score for all the folds that are used in this study and also has better accuracy. Besides that ROC curve also indicates Random Forest has the largest area under the curve in comparison to all other models for the training data set and for the test data set, C5.0 has the largest area under the curve. Classification and Regression Tree, Gradient Boosting Machine, and C5.0 also performed very well in predicting malnutrition but among them, Random Forest can be taken as the best classifier for predicting malnutrition in women. Naïve Bayes and Logistic Regression can predict malnutrition as well but they are quite low in accuracy for predicting the nutritional status of women in Bangladesh. This study advocated that all Decision Tree based classifiers evaluate the malnutrition of women very well but among them, Random Forest was implemented very well. Hence, the Random Forest system be utilized as the best prediction-based system for predicting malnutrition in women in Bangladesh because it outperforms the other models employed in the study.

This research will aid healthcare practitioners and regulators in developing a system for adopting necessary interventions along with care practices in order to minimize serious problems and the load on the healthcare system. Furthermore, the study demonstrates how the ML technique may be used to better forecast the fundamental causes of women’s malnourishment, in addition to other population health consequences. This could lead to a better knowledge of women’s nutritional condition and the establishment of more successful programs to improve womanhood nutrition in the country. There is a need for initiatives and policies that focus on women of various ages and locations, as well as improving women’s socioeconomic well-being in Bangladesh. As a result, a model that takes into account the basic types of risk would aid in the prevention and control of female malnutrition. The ethical ramifications of using machine learning algorithms in healthcare, particularly in countries with low or middle incomes with less developed regulatory systems, include concerns with patient information privacy, bias reduction, informed consent, and the requirement for rules and regulations to guarantee responsible and fair application of AI in healthcare. Certain ethical issues must be addressed for machine learning to be successfully and morally integrated into healthcare systems.

This study solely focused on the women population and was conducted with the cross-sectional BDHS data. Therefore, the ML models might not perform the same on the other groups of the population. This study faced constraints in terms of computational resources and dataset complexities, even though it acknowledged the potential benefits of incorporating additional machine learning models like Support Vector Machines (SVM), Artificial Neural Networks (ANN), and exploring alternative feature selection techniques. The restricted processing capability hindered the comprehensive testing of many models, and the size and characteristics of the dataset made it difficult to use some feature selection techniques efficiently within the parameters of this study. Because of this, the current work concentrated on a particular set of feature selection techniques and machine learning models. To overcome these constraints and improve the analysis’s depth and robustness, future efforts will focus on increasing computational power and improving dataset preprocessing to include a larger range of models and feature selection strategies.

## Data Availability

The data in this article comes from the DHS Program database. This data can be found here: https://dhsprogram.com/Data/. The data sets used during the current study are available from the corresponding author upon reasonable request. Please contact the corresponding author for further information.

## References

[1] Black RE, Victora CG, Walker SP, Bhutta ZA, Christian P, De Onis M (2013). Maternal and child undernutrition and overweight in low-income and middle-income countries. Lancet..

[2] National Institute of Population Research and Training (NIPORT). Mitra and Associates, and ICF International. NIPORT, mitra and associates, and icf international. Dhaka, Bangladesh, and Rockville, Maryland, USA: Bangladesh Demographic and Health Survey. 2014.

[3] Islam MM, Rahman MJ, Islam MM, Roy DC, Ahmed NF, Hussain S (2022). Application of machine learning based algorithm for prediction of malnutrition among women in Bangladesh. Int J Cogn Comput Eng..

[4] Kc B (2019). Factors responsible for non-communicable diseases among Bangladeshi adults. Biomed J Sci Tech Res..

[5] Nyberg ST, Batty GD, Pentti J, Virtanen M, Alfredsson L, Fransson EI (2018). Obesity and loss of disease-free years owing to major non-communicable diseases: a multicohort study. Lancet Public health..

[6] Rahman A, Sathi NJ (2021). Sociodemographic risk factors of being underweight among ever-married Bangladeshi women of reproductive age: a multilevel analysis. Asia Pac J Public Health..

[7] Rawal LB, Kanda K, Mahumud RA, Joshi D, Mehata S, Shrestha N (2018). Prevalence of underweight, overweight and obesity and their associated risk factors in Nepalese adults: Data from a Nationwide Survey, 2016. PLoS ONE..

[8] Boutari C, Pappas PD, Mintziori G, Nigdelis MP, Athanasiadis L, Goulis DG (2020). The effect of underweight on female and male reproduction. Metabolism..

[9] Khan MN, Rahman MM, Shariff AA, Rahman MM, Rahman MS, Rahman MA (2017). Maternal undernutrition and excessive body weight and risk of birth and health outcomes. Arch Public Health..

[10] Melchor I, Burgos J, Del Campo A, Aiartzaguena A, Gutiérrez J, Melchor JC (2019). Effect of maternal obesity on pregnancy outcomes in women delivering singleton babies: a historical cohort study. J Perinat Med..

[11] Ismail SR, Mehmood A, Rabiah N, Abu-sulaiman RM, Kabbani MS (2021). Impact of the nutritional status of children with congenital heart diseases on the early post-operative outcome. Egypt Pediatr Assoc Gaz..

[12] Pal A, Manna S, Dalui R, Mukhopadhyay R, Dhara PC (2021). Undernutrition and associated factors among children aged 5–10 years in West Bengal, India: a community-based cross-sectional study. Egypt Pediatr Assoc Gaz..

[13] Ahmad D, Afzal M, Imtiaz A (2020). Effect of socioeconomic factors on malnutrition among children in Pakistan. Futur Bus J..

[14] Ekholuenetale M, Tudeme G, Onikan A, Ekholuenetale CE (2020). Socioeconomic inequalities in hidden hunger, undernutrition, and overweight among under-five children in 35 sub-Saharan Africa countries. J Egypt Public Health Assoc..

[15] Hagos S, Hailemariam D, WoldeHanna T, Lindtjørn B. Spatial heterogeneity and risk factors for stunting among children under age five in Ethiopia: a Bayesian geo-statistical model. PLoS ONE. 2017;12(2):0170785. 10.1371/journal.pone.0170785 PMC5295674 28170407

[16] Thompson DS, Younger-Coleman N, Lyew-Ayee P, Greene LG, Boyne MS, Forrester TE (2017). Socioeconomic factors associated with severe acute malnutrition in Jamaica. PLoS ONE..

[17] Ekholuenetale M, Barrow A, Ekholuenetale CE, Tudeme G (2020). Impact of stunting on early childhood cognitive development in Benin: evidence from Demographic and Health Survey. Egypt Pediatr Assoc Gaz..

[18] Rahman MS, Mushfiquee M, Masud MS, Howlader T (2019). Association between malnutrition and anemia in under-five children and women of reproductive age: Evidence from Bangladesh Demographic and Health Survey 2011. PLoS ONE..

[19] Abedin MM, Haque ME, Sabiruzzaman M, Al Mamun ASM, Hossain MG (2019). Multinomial logistic regression analysis of factors influencing malnutrition of non-pregnant married women in Bangladesh: Evidence from Bangladesh Demographic and Health Survey-2014.

[20] Hossain MM, Islam MR, Sarkar ASR, Khan MMA, Taneepanichskul S (2018). Prevalence and determinants risk factors of underweight and overweight among women in Bangladesh. Obes Med..

[21] Khanam R, Lee ASC, Ram M, Quaiyum M, Begum N, Choudhury A (2018). Levels and correlates of nutritional status of women of childbearing age in rural Bangladesh. Public Health Nutr..

[22] Tanwi TS, Chakrabarty S, Hasanuzzaman S (2019). Double burden of malnutrition among ever-married women in Bangladesh: a pooled analysis. BMC Women’s Health..

[23] Kumar D, Goel N, Mittal PC, Misra P (2006). Influence of infant-feeding practices on nutritional status of under-five children. Indian J Pediatr..

[24] Frongillo EA, de Onis M, Hanson KM (1997). Socioeconomic and demographic factors are associated with worldwide patterns of stunting and wasting of children. J Nutr..

[25] Ngiam KY, Khor W (2019). Big data and machine learning algorithms for health-care delivery. Lancet Oncol..

[26] Mitchell TM, Mitchell TM. Machine learning, vol 1. McGraw-hill New York; 1997.

[27] Alghamdi M, Al-Mallah M, Keteyian S, Brawner C, Ehrman J, Sakr S (2017). Predicting diabetes mellitus using SMOTE and ensemble machine learning approach: The Henry Ford ExercIse Testing (FIT) project. PLoS ONE..

[28] Jaiswal M, Srivastava A, Siddiqui TJ. Machine learning algorithms for anemia disease prediction. In: Recent trends in communication, computing, and electronics. Springer; 2019. p. 463–469.

[29] Khan JR, Chowdhury S, Islam H, Raheem E (2019). Machine learning algorithms to predict the childhood anemia in Bangladesh. J Data Sci..

[30] Hsieh CH, Lu RH, Lee NH, Chiu WT, Hsu MH, Li YCJ (2011). Novel solutions for an old disease: diagnosis of acute appendicitis with random forest, support vector machines, and artificial neural networks. Surgery..

[31] Louridi N, Douzi S, El Ouahidi B (2021). Machine learning-based identification of patients with a cardiovascular defect. J Big Data..

[32] Laatifi M, Douzi S, Bouklouz A, Ezzine H, Jaafari J, Zaid Y (2022). Machine learning approaches in Covid-19 severity risk prediction in Morocco. J Big Data..

[33] Rezaeijo SM, Ghorvei M, Abedi-Firouzjah R, Mojtahedi H, Zarch HE (2021). Detecting COVID-19 in chest images based on deep transfer learning and machine learning algorithms. Egypt J Radiol Nucl Med..

[34] Meng XH, Huang YX, Rao DP, Zhang Q, Liu Q (2013). Comparison of three data mining models for predicting diabetes or prediabetes by risk factors. Kaohsiung J Med Sci..

[35] Nibareke T, Laassiri J (2020). Using Big Data-machine learning models for diabetes prediction and flight delays analytics. J Big Data..

[36] Sharma T, Shah M (2021). A comprehensive review of machine learning techniques on diabetes detection. Vis Comput Ind Biomed Art..

[37] Yu W, Liu T, Valdez R, Gwinn M, Khoury MJ (2010). Application of support vector machine modeling for prediction of common diseases: the case of diabetes and pre-diabetes. BMC Med Inf Decis Making..

[38] Islam MM, Rahman MJ, Roy DC, Tawabunnahar M, Jahan R, Ahmed NF (2021). Machine learning algorithm for characterizing risks of hypertension, at an early stage in Bangladesh. Diabetes Metab Syndr Clin Res Rev..

[39] Islam Pollob SA, Abedin MM, Islam MT, Islam MM, Maniruzzaman M (2022). Predicting risks of low birth weight in Bangladesh with machine learning. PLoS ONE..

[40] Borson NS, Kabir MR, Zamal Z, Rahman RM. Correlation analysis of demographic factors on low birth weight and prediction modeling using machine learning techniques. In: 2020 Fourth World Conference on Smart Trends in Systems, Security and Sustainability (WorldS4). IEEE; 2020. p. 169–173.

[41] Eliyati N, Faruk A, Kresnawati ES, Arifieni I. Support vector machines for classification of low birth weight in Indonesia. In: Journal of Physics: Conference Series, vol 1282. IOP Publishing; 2019. p. 012010.

[42] Faruk A, Cahyono ES (2018). Prediction and classification of low birth weight data using machine learning techniques. Indones J Sci Technol..

[43] Talukder A, Ahammed B (2020). Machine learning algorithms for predicting malnutrition among under-five children in Bangladesh. Nutrition..

[44] Bitew FH, Nyarko SH, Potter L, Sparks CS (2020). Machine learning approach for predicting under-five mortality determinants in Ethiopia: evidence from the 2016 Ethiopian Demographic and Health Survey. Genus..

[45] Khare S, Kavyashree S, Gupta D, Jyotishi A (2017). Investigation of nutritional status of children based on machine learning techniques using Indian demographic and health survey data. Procedia Comput Sci..

[46] Mukuku O, Mutombo AM, Kamona LK, Lubala TK, Mawaw PM, Aloni MN, et al. Predictive model for the risk of severe acute malnutrition in children. J Nutr Metab. 2019. 10.1155/2019/4740825 PMC6636463 31354989

[47] Shahriar MM, Iqubal MS, Mitra S, Das AK. A Deep Learning Approach to Predict Malnutrition Status of 0-59 Month’s Older Children in Bangladesh. In: 2019 IEEE International Conference on Industry 4.0, Artificial Intelligence, and Communications Technology (IAICT). IEEE; 2019. p. 145–149.

[48] Markos Z, Doyore F, Yifiru M, Haidar J (2014). Predicting under nutrition status of under-five children using data mining techniques: the case of 2011 Ethiopian Demographic and Health Survey. J Health Med Inform..

[49] Reis R, Peixoto H, Machado J, Abelha A (2017). Machine Learning in Nutritional Follow-up Research. Open Comput Sci..

[50] Momand Z, Mongkolnam P, Kositpanthavong P, Chan JH. Data Mining Based Prediction of Malnutrition in Afghan Children. In: 2020 12th International Conference on Knowledge and Smart Technology (KST), 2020. p. 12–17. 10.1109/KST48564.2020.9059388.

[51] Rahman SJ, Ahmed NF, Abedin MM, Ahammed B, Ali M, Rahman MJ (2021). Investigate the risk factors of stunting, wasting, and underweight among under-five Bangladeshi children and its prediction based on machine learning approach. PLoS ONE..

[52] National Institute of Population Research and Training (NIPORT), and ICF. Bangladesh Demographic and Health Survey 2017-18. Dhaka and Rockville: NIPORT and ICF; 2020.

[53] Who O (2000). preventing and managing the global epidemic. Geneva. WHO Tech Rep Ser..

[54] Ahmed KY, Rwabilimbo AG, Abrha S, Page A, Arora A, Tadese F (2020). Factors associated with underweight, overweight, and obesity in reproductive age Tanzanian women. PLoS ONE..

[55] Han J, Kamber M, Pei J. Data mining concepts and techniques. 3rd ed. University of Illinois at Urbana-Champaign Micheline Kamber Jian Pei Simon Fraser University; 2012.

[56] Cover TM (1965). Geometrical and statistical properties of systems of linear inequalities with applications in pattern recognition. IEEE Trans Electron Comput..

[57] Breiman L. Classification and regression trees. Routledge; 2017.

[58] Quinlan JR. Data mining tools See5 and C5. 0. 2004. https://api.semanticscholar.org/CorpusID:59843478. Accessed 29 Mar 2023.

[59] Gutierrez DD. Machine learning and data science: an introduction to statistical learning methods with R. Technics Publications; 2015.

[60] Breiman L (2001). Random forests. Mach Learn..

[61] Hastie T, Tibshirani R, Friedman JH, Friedman JH. The elements of statistical learning: data mining, inference, and prediction, vol 2. Springer; 2009.

[62] Friedman JH (2001). Greedy function approximation: a gradient boosting machine. Ann Stat..

[63] Brownlee J. Machine learning mastery with R: Get started, build accurate models and work through projects step-by-step. Machine Learning Mastery; 2016.

[64] Landis JR, Koch GG (1977). The measurement of observer agreement for categorical data. Biometrics..

